# GalNAc-Lipid nanoparticles enable non-LDLR dependent hepatic delivery of a CRISPR base editing therapy

**DOI:** 10.1038/s41467-023-37465-1

**Published:** 2023-05-15

**Authors:** Lisa N. Kasiewicz, Souvik Biswas, Aaron Beach, Huilan Ren, Chaitali Dutta, Anne Marie Mazzola, Ellen Rohde, Alexandra Chadwick, Christopher Cheng, Sara P. Garcia, Sowmya Iyer, Yuri Matsumoto, Amit V. Khera, Kiran Musunuru, Sekar Kathiresan, Padma Malyala, Kallanthottathil G. Rajeev, Andrew M. Bellinger

**Affiliations:** 1 Verve Therapeutics, 201 Brookline Avenue, Suite 601, Boston, MA 02215 USA; 2 Division of Cardiovascular Medicine, Department of Medicine, Perelman School of Medicine at the University of Pennsylvania, Philadelphia, PA USA

**Keywords:** Drug delivery, Gene therapy, Nucleic-acid therapeutics, Cardiovascular diseases

## Abstract

Lipid nanoparticles have demonstrated utility in hepatic delivery of a range of therapeutic modalities and typically deliver their cargo via low-density lipoprotein receptor-mediated endocytosis. For patients lacking sufficient low-density lipoprotein receptor activity, such as those with homozygous familial hypercholesterolemia, an alternate strategy is needed. Here we show the use of structure-guided rational design in a series of mouse and non-human primate studies to optimize a GalNAc-Lipid nanoparticle that allows for low-density lipoprotein receptor independent delivery. In low-density lipoprotein receptor-deficient non-human primates administered a CRISPR base editing therapy targeting the *ANGPTL3* gene, the introduction of an optimized GalNAc-based asialoglycoprotein receptor ligand to the nanoparticle surface increased liver editing from 5% to 61% with minimal editing in nontargeted tissues. Similar editing was noted in wild-type monkeys, with durable blood ANGPTL3 protein reduction up to 89% six months post dosing. These results suggest that GalNAc-Lipid nanoparticles may effectively deliver to both patients with intact low-density lipoprotein receptor activity as well as those afflicted by homozygous familial hypercholesterolemia.

## Abstract (web summary)

Kasiewicz et al. describe a structure-guided rational design approach to optimize a new GalNAc-Lipid nanoparticle that enables delivery of a base editing therapy in both low-density lipoprotein receptor-deficient and wild-type nonclinical models.

## Introduction

For a range of important conditions, nucleic acid delivery to the liver has the potential to directly target disease-causing pathways. Lipid nanoparticles (LNPs) have emerged as a preferred strategy for hepatic delivery as compared to viral vectors, which have been noted to integrate into the host DNA, demonstrate prolonged expression, and elicit an immunologic response^1–6^. After intravenous infusion of a standard LNP, the accumulation of apolipoprotein E (ApoE) on the surface leads to uptake primarily by hepatocytes via low-density lipoprotein receptor (LDLR)-mediated endocytosis^7^.

An alternative approach to hepatic LNP uptake mediated by endogenous ApoE/LDLR binding is to use a multi-valent *N*-acetylgalactosamine (GalNAc) targeting ligand, which allows for uptake via the asialoglycoprotein receptor (ASGPR) pathway^8^. Delivery via ASGPR has several favorable properties: the receptor is highly expressed in the liver but not other tissues, leads to rapid endocytosis of the medicine when bound by GalNAc, and is rapidly recycled to the hepatocyte surface^1^. Although a GalNAc-LNP approach has not previously been described in the context of a genome editing medicine, this approach has proven useful in the hepatic delivery of other therapeutic technologies, including the siRNA inclisiran (approved for treatment for hypercholesterolemia) and the antisense oligonucleotide (ASO) eplontersen (in advanced stages of clinical development for the treatment of amyloidosis)^9,10^.

Beyond siRNA and ASO therapeutic modalities, CRISPR-based therapies directed toward the liver have the potential to enable DNA modifications that allow for permanent inactivation of disease-causing genes after a single dose^11,12^. Here, we test the hypothesis that a structure-guided rational design approach would allow for the development of a GalNAc-LNP capable of delivering a CRISPR base editing therapeutic via LDLR-independent pathways. This delivery approach is of particular interest for the potential treatment of patients with homozygous familial hypercholesterolemia (HoFH), a rare genetic disorder characterized by severe hepatic LDLR-deficiency^13^. In contrast to prior in vivo preclinical studies of siRNA and ASOs conducted only in mouse models, we extend our results into both wild-type NHPs and a newly developed NHP model of LDLR-deficiency^14–16^.

## Results

### Structure-guided rational design of GalNAc-Lipids

**Fig. 1:**
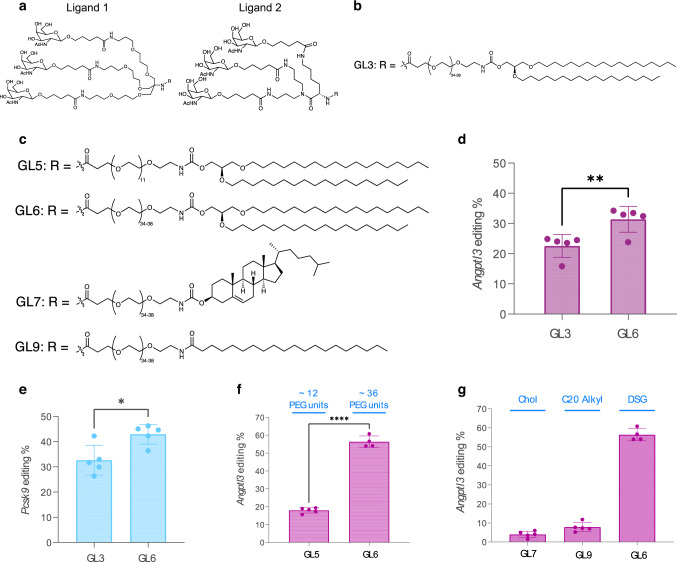
Structures and initial screen of GalNAc-Lipids. **a** Structure of ligand Designs 1 and 2. **b** Structure of R moiety for GL3, which uses ligand Design 1. **c** Structures of R moiety for GL5, GL6, GL7, and GL9. These structures utilize ligand Design 2 but differ in their lipid anchors and polyethylene glycol (PEG) spacer lengths. **d** GalNAc-LNPs constituted with 0.05 mol % GL3 (ligand Design 1 – Table S1, entry 2) or 0.05 mol % GL6 (ligand Design 2 – Table S1, entry 1) were prepared via the in-lipid mixing method and were administered to female 8-10 week old *Ldlr* ^–/–^ mice via injection into the retro-orbital sinus at a dose of 0.1 mg/kg. GL3 and GL6 differ only in the ligand design; the PEG spacer and lipid chain are the same. Ligand 2-based GL6 GalNAc-LNPs achieved higher *Angptl3* liver editing when mice were administered GalNAc-LNPs encapsulating mouse-specific *Angptl3* guide RNA and ABE8.8 mRNA. Data was analyzed with a two-tailed unpaired T test, *p* = 0.0086 (df=8, mean difference = 8.9%, 95% confidence interval 2.9–14.7%). **e** GalNAc-LNPs constituted with 0.5 mol % GL3 (Table S1, entry 4) or GL6 (Table S1, entry 3) were prepared via the post-addition method and were administered to female *Ldlr* ^–/–^ mice via injection into the retro-orbital sinus at a dose of 0.25 mg/kg. Ligand Design 2-based GL6 GalNAc-LNPs achieved higher *Pcsk9* liver editing, when mice were administered GalNAc-LNPs encapsulating mouse-specific *Pcsk9* guide RNA and ABE8.8 mRNA (*N* = 5). Data were analyzed with a two-tailed unpaired T test, *p* = 0.01 (df = 8, mean difference = 10.3%, 95% confidence interval 2.9–17.6%). **f** GalNAc-LNPs formulated with the longer PEG spacer of GL6 (Table S1, entry 6) achieved higher editing of *Angptl3* than the GalNAc-LNPs with the shorter PEG spacer of GL5 (Table S1 entry 5) at 0.3 mg/kg in female *Ldlr* ^–/–^ mice. Data were analyzed with a two-tailed unpaired T test, *p* < 0.0001 (df = 7, mean difference = 38.5%, 95% confidence interval 34.7–42.2%). **g** Modulation of the lipid tail hydrophobicity in GL7 and GL9 (Table S1, entries 7 and 8) with cholesteryl (Chol) and arachidoyl (C20) moieties was unable to improve the editing efficiency of GalNAc-LNPs in female *Ldlr* ^–/–^ mice compared to the 1,2-O-dioctadecyl-sn-glyceryl (DSG)-based GL6 (Table S1, entry 6) at 0.3 mg/kg. Data are presented as mean values + /- standard deviation, and individual data points for each animal are displayed (*n* = 5). * denotes *p* < 0.05, ** denotes *p* value <0.01, **** denotes *p* < 0.0001. Source data are provided as a Source Data file.

To develop a GalNAc-LNP capable of delivering a CRISPR base editing therapy, we undertook structure-guided rational design of GalNAc-based ASGPR ligands. Proximity and tethering of the sugar moieties in the multi-valent ligand design are critical for efficient recognition and binding to ASGPR, thus facilitating uptake into hepatocytes^8^. To ensure both sugar proximity and optimal tethering, three GalNAc units were covalently attached to two different scaffolds through the multiatom spacing between each sugar moiety and the scaffold. We thus obtained ligand Design 1 and Design 2 (Fig. 1a). Design 1 is akin to a clinically validated ligand design and utilizes a TRIS-scaffold^8^. The nitrogen atom of the TRIS-scaffold enables covalent attachment of the ligand to a lipid anchor, thus allowing for incorporation into an LNP. In ligand Design 2, a lysine-based scaffold was designed to covalently attach three sugar units and the lipid anchor. Design 2 has potential advantages over Design 1 due to the simplicity and ease of manufacturing the trivalent GalNAc ligand and the GalNAc-Lipid at scale. The TRIS-based trivalent ligand Design 1, as seen in GalNAc-Lipid GL3 (Fig. 1b), was replaced with the lysine-based trivalent ligand in Design 2 to yield GalNAc-Lipid GL6 (Fig. 1c) comprising the same PEG-spacer and lipid anchor. The Design-2 based GalNAc-Lipids GL6, GL7, and GL9 were designed and synthesized to evaluate the impact of: (a) PEG-spacing between the ligand and lipid anchor, (b) the hydrophobic packing of the lipid chain in the LNP during the formulation of GalNAc-LNP, and (c) the effect of GalNAc-Lipid structural variations on ASGPR recognition and binding to facilitate uptake into hepatocytes.

To compare the efficiency of various GalNAc-LNPs to deliver to the liver in vivo via non-LDLR-dependent pathways, a series of screening experiments were performed in *Ldlr* ^−/−^ mice. GalNAc-LNPs (Table S1) were formulated with an adenine base editor 8.8-m (ABE8.8) mRNA and a guide RNA (gRNA) targeting the mouse *Angptl3* or *Pcsk9* genes—well-validated therapeutic targets for the treatment of hypercholesterolemia. The first such experiment tested GalNAc-Lipids GL3 and GL6, which share the same PEG spacer and lipid anchor but differ in the ligand design. GalNAc-LNPs constituted with 0.05 mol % GL3 or 0.05 mol % GL6 were administered to *Ldlr*^−/−^ mice (*N* = 5 for each treatment group) at a dose of 0.1 mg/kg. Five to ten days following treatment, scheduled necropsy was performed, and liver editing was assessed via targeted amplicon sequencing. An increased rate of editing was observed for animals treated with the GalNAc-LNP containing GL6, mean 31% versus 23% respectively (*p* value = 0.0086), as noted in Fig. 1d. A similar pattern was noted in a second experiment comparing GL3 versus GL6 with a guide RNA targeting the *Pcsk9* gene, where *Ldlr*^−/−^ mice (*N* = 5 for each treatment group) were treated at a dose of 0.25 mg/kg. Mean liver editing was again significantly higher in mice treated with the GalNAc-LNP containing GL6 as compared to GL3, mean 43% versus 33%, respectively (*p* value = 0.01, Fig. 1e).

Having prioritized the head group of ligand Design 2, we next turned our attention to optimizing the lipid anchor and spacer design. We replaced the average 36-unit PEG linker of GL6 with a 12-unit PEG linker to obtain GL5 (Fig. 1c) to evaluate the effect of spacing between the nanoparticle and the surface-bound ligand. The potency of an LNP with the longer PEG spacer of GL6 as compared to the shorter PEG spacer of GL5 was assessed in an *Ldlr*^−/−^ mouse experiment, with both formulations including the same adenine base editor and guide RNA targeting *Angptl3*. After dosing 4-5 animals in each treatment group at a dose of 0.3 mg/kg, the degree of *Angptl3* liver editing was observed to be significantly higher with the GL6 formulation as compared to GL5, mean 56% versus 18% respectively (p-value <0.0001; Fig. 1f, Supplementary Fig. 1), and the longer PEG spacer was thus selected for subsequent studies.

**Fig. 2:**
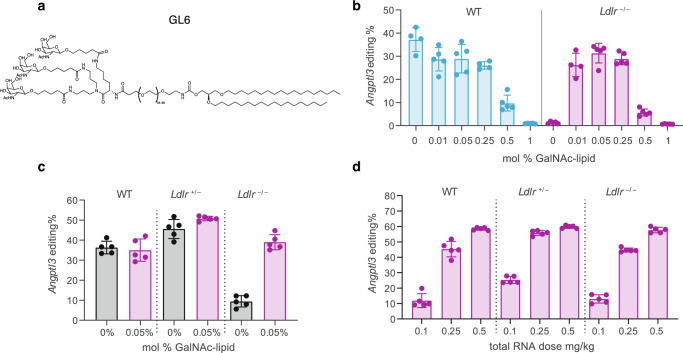
GalNAc-Lipid optimization in LDLR-deficient mouse models. **a** GalNAc-Lipid GL6 comprising a PEG spacer and 1,2-di-*O*-octadecyl-*sn*-glyceryl lipid anchor; **b** titration of the surface density of GalNAc-Lipid demonstrated a low density near 0.05 mol % of GalNAc-Lipid optimally rescues liver editing in female 8–10 week old *Ldlr* ^–/–^ mice while preserving editing in wild type (WT) mice at an RNA dose of 0.1 mg/kg (Table S1, entries 9–14); **c** LNPs constituted with 0.05 mol % GL6 maintained *Angptl3* base editing in female WT and *Ldlr* ^+/–^ mice and rescued base editing in *Ldlr* ^–/–^ mice in vivo at 0.25 mg/kg; **d** demonstration of near-identical dose response of liver *Angptl3* editing using the optimized GalNAc-LNPs (constituted with 0.05 mol % GL6) in three genotypes: WT, *Ldlr* ^+/–^, and *Ldlr* ^–/–^. Data are presented as mean values + /- standard deviation, and individual data points for each animal are displayed (*n* = 5). Source data are provided as a Source Data file.

The residence time of the ligand on the LNP surface is also critical for ASGPR recognition and binding. We evaluated three different lipid anchors, namely 1,2-*O*-dioctadecyl-*sn*-glyceryl (DSG), cholesteryl (Chol), and arachidoyl (C20). Different anchor structures can affect how long the GalNAc-Lipid remains incorporated into the nanoparticle before being shed or sheared away, thus potentially affecting the ability to bind ASGPR successfully. These lipid anchors were attached to ligand Design 2 via an average 36-unit PEG linker to generate GL7 and GL9 (Fig. 1c). GalNAc-LNPs constituted with each lipid anchor were assessed in an *Ldlr*^−/−^ mouse experiment, with all formulations including the same adenine base editor and guide RNA targeting *Angptl3*. *Angptl3* gene editing (Fig. 1g) and Angptl3 protein knockdown (Fig. S1) in *Ldlr*^−/−^ mice treated with 0.3 mg/kg demonstrated that the DSG lipid anchor was considerably more potent versus the cholesterol and C20 lipid anchors with the same PEG linker (Fig. 1g), mean 56% versus 4% and 8% respectively (*p*-value <0.0001). GL6 (Fig. 2a) was thus identified as a high-performing GalNAc-Lipid to facilitate robust LDLR-independent delivery of LNPs to LDLR-deficient hepatocytes in vivo.

### Optimization of GalNAc-LNPs

For any given selected GalNAc-Lipid, several strategies for incorporation in the LNP during the manufacturing process warrant consideration. Initial attempts to incorporate the GalNAc-Lipid after LNP formulation resulted in a non-uniform distribution of GalNAc-Lipid in the drug product, as assessed based on a lectin binding assay (Supplementary Fig. 2). As an alternative, we formulated the GalNAc-LNP by mixing the GalNAc-Lipid with other lipid excipients prior to LNP particle formation and generated stable particles which produced similar efficacy in mice (Supplementary Fig. 3) and allowed for efficient scale-up to larger batch sizes.

To optimize the surface density of GalNAc-ligand required for efficient ASGPR recognition, molar percentages of GalNAc-Lipid ranging from 0 to 1% were assessed in vivo in both wild-type (WT) and *Ldlr* ^−/−^ mice (Fig. 2b). As expected, minimal *Angptl3* editing was observed in *Ldlr* ^−/−^ mice using an LNP with 0% GalNAc-Lipid, with mean editing of 1.3%, but as little as 0.01 mol % GalNAc-Lipid substantially rescued editing to a mean of 26.3% and inclusion of 0.05 mol % produced the highest mean editing of 31.4%. Increasing the density of GalNAc-ligand on the LNP surface decreased efficacy in both WT and *Ldlr* ^−/−^ mice. This titration of ligand surface density indicated crowding the LNP surface with ligand is detrimental to the ASGPR-mediated uptake of GalNAc-LNP.

To study the potency of standard and GalNAc-LNPs across the full range of normal LDLR activity, heterozygous deficiency, and homozygous deficiency, an additional mouse screening experiment was conducted. We dosed *Ldlr* ^−/−^, *Ldlr* ^*+*/–^, and WT mice with 0.25 mg/kg of LNPs formulated with and without 0.05 mol% GalNAc-Lipid GL6. Editing was similar between standard and GalNAc-LNPs, with 36% vs 35% respectively in WT and 46% vs 51% in *Ldlr* ^*+*/–^ mice. GalNAc-Lipid was able to rescue editing in *Ldlr* ^−/−^ mice, increasing *Angptl3* editing from 9% to 39% (*p*-value <0.0001 Fig. 2c, Supplementary Fig. 4). The inclusion of GalNAc-Lipid thus enabled similar editing efficiency in all three *Ldlr* genotypes, which was further seen in a dose response at 0.1, 0.25, and 0.5 mg/kg doses (Fig. 2d).

### Development of an LDLR-deficient non-human primate model

**Fig. 3:**
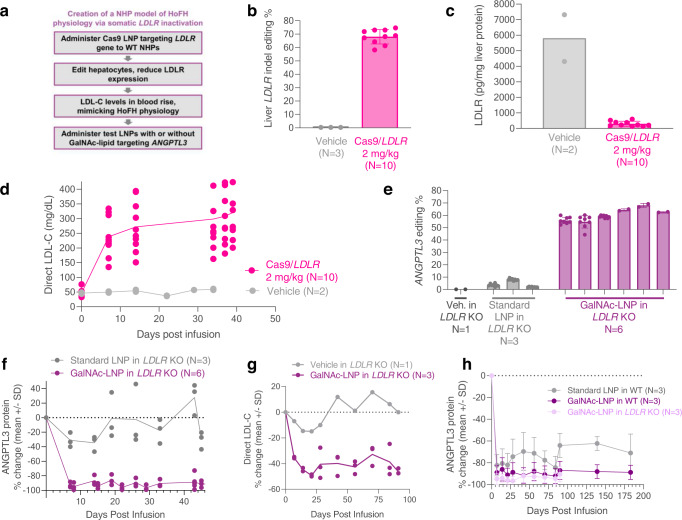
Demonstration of adenine base editing by GalNAc-LNPs targeting *ANGPTL3* in the liver of a somatic LDLR-deficient NHP model. **a** Schematic detailing the creation of the somatic low-density lipoprotein receptor (LDLR) deficient/knockout (KO) model in non-human primates (NHPs) using CRISPR-Cas9 lipid nanoparticles (LNPs). 2–3-year-old wild-type (WT) male cynomolgus NHPs were treated with 2 mg/kg of SpCas9 dual-gRNA LNPs targeting the *LDLR* gene, editing and disrupting *LDLR* in the liver. **b** Liver biopsy demonstrated editing of 68% of all *LDLR* alleles in a liver biopsy. **c** LDLR protein levels assayed by ELISA on a second liver biopsy were markedly reduced by 95%, and (**d**) blood low-density lipoprotein cholesterol (LDL-C) increased from ~50 mg/dL to ~300 mg/dL. LNPs without GalNAc-Lipid were not effective in *LDLR*-deficient NHPs, yielding (**e**) 4.5% *ANGPTL3* editing and (**f**) minimal or no ANGPTL3 protein reductions at a 2 mg/kg dose. GalNAc-LNPs at a 2 mg/kg total RNA dose and with 0.05 mol % GL6 resulted in rescue of (**e**) high-efficiency liver *ANGPTL3* editing and (**f**) durable 89% reduction in blood ANGPTL3 protein out to three months. In panel (**e**), each column represents an NHP, and editing results reflect liver biopsy (1-2) or necropsy (8) samples. In the homozygous familial hypercholesterolemia HoFH NHP model with markedly elevated baseline LDL-C levels of ~300 mg/dL, editing of *ANGPTL3* with GalNAc-LNPs (**g**) lowered LDL-C (~35%, or ~100 mg/dL in absolute terms) out to three months. **h** Comparison of blood ANGPTL3 reductions out to six months in WT NHPs dosed with either standard LNPs or GalNAc-LNPs at 2 mg/kg. Data are presented as mean values + /- standard deviation (where error bars are present) and individual data points for each animal are displayed in panels (**b**–**g**). Panels (**d**), (**f**), and (**g**) have the means plotted as a line along with the individual points. Panel (**h**) averages three ANGPTL3 protein values from three distinct NHPs and the data are presented as mean values + /- standard deviation. Source data are provided as a Source Data file.

Based on results in mouse studies suggesting that optimized GalNAc-LNPs have the potential to enable efficient delivery of a CRISPR base-editing medicine targeting *ANGPTL3* via non-LDLR-dependent pathways, we next sought to further evaluate this in a cynomolgus monkey non-human primate (NHP) model. Because LDLR-deficient NHPs—which would serve as a preclinical model of HoFH—are not readily available, such a model was newly developed (Fig. 3a). Two gRNAs targeting different locations 22 base pairs apart in the *LDLR* gene (with an expected 34 base pair deletion) were co-formulated with *Streptococcus pyogenes* Cas9 (SpCas9) mRNA in a standard LNP formulation and intravenously administered to 10 WT NHPs at a 2 mg/kg dose, with an additional 3 NHPs treated with a vehicle control. This approach led to a mean editing of the *LDLR* gene of 68% in animals treated with the *LDLR* guides versus less than 0.4% in those treated with vehicle control (Fig. 3b). As expected, most of the observed edits were the deletion of 31–40 base pairs (Table S3). Confirmation of disruption of *LDLR* was determined via quantification of liver protein in specimens obtained via biopsy 19 days postdosing. Mean LDLR values of 301 versus 5810 pg/mL (*p* < 0.0001) were observed for those treated with the gRNAs targeting *LDLR* and control, respectively, corresponding to a 95% reduction (Fig. 3c). As expected, given the central role of the LDLR in clearing LDL-cholesterol (LDL-C) from the circulation, a marked increase in LDL-C concentrations was observed in treated NHPs, increasing from approximately 50 mg/dL at time of infusion to over 300 mg/dL after 40 days (Fig. 3d). Taken together, these data indicated successful generation of a liver somatic LDLR-deficient NHP model.

### Potency and tolerability of standard and GalNAc-LNPs in LDLR-deficient NHPs in vivo

We next tested both standard LNPs and GalNAc-LNPs for the ability to deliver gene editing cargo in LDLR-deficient NHPs. In 3 LDLR-deficient NHPs treated with standard LNPs (not formulated with GalNAc-Lipid) at a dose of 2 mg/kg (Table S2), minimal liver *ANGPTL3* editing was observed at the target site (mean 4.5%), corresponding to a modest reduction in blood ANGPTL3 protein of 13% (Fig. 3e-f).

By contrast, mean liver *ANGPTL3* editing of 61% was observed in the 6 NHPs treated with the GalNAc-LNPs (Table S2), corresponding to a reduction in blood ANGPTL3 protein of 89% (Fig. 3e-f). Blood LDL-C also fell by 35%, a reduction stable for three months after treatment (Fig. 3g). In absolute terms, this was a ~100 mg/dL reduction in LDL-C in the HoFH NHP model. Liver safety monitoring noted transient increases in alanine aminotransferase (ALT) and aspartate aminotransferase (AST) for GalNAc-LNP treated NHPs, though for AST this was not statistically different from vehicle-treated controls (Fig. S5). Maximal mean ALT values of 480 U/L were seen 48 hours after treatment with GalNAc-LNPs and a maximal mean AST value of 537 U/L was observed 6 hours after treatment. Both ALT and AST normalized to baseline values by 14 days after treatment.

Assessment of circulating cytokines that reflect innate immune system activation – tumor necrosis factor-alpha (TNFα) and monocyte chemoattractant protein-1 (MCP-1) – noted a similar pattern with values transiently increasing and returning to baseline by 7 days post infusion (Supplementary Fig. 5).

### Potency and tolerability of standard and GalNAc-LNPs in wild-type NHPs in vivo

**Fig. 4:**
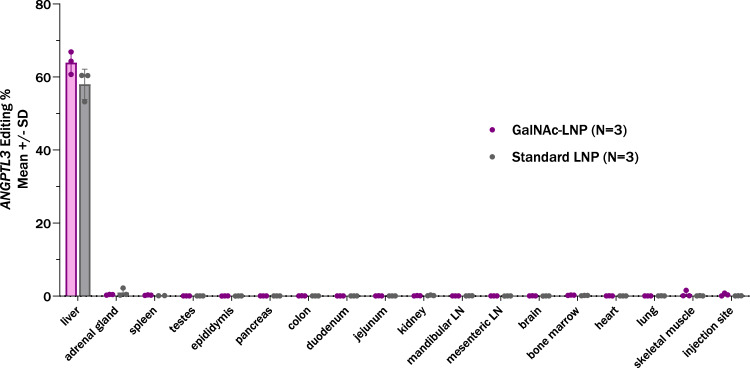
Hepatic and extra-hepatic editing of *ANGPTL3* following treatment with a GalNAc-LNP or standard LNP. Targeted amplicon sequencing of the *ANGPTL3* target site was performed in tissue samples collected at necropsy following dosing with standard lipid nanoparticles (LNPs) (*N* = 3) and GalNAc-LNPs (*N* = 3) at a 2 mg/kg dose. Biodistribution and liver editing of GalNAc-LNPs and standard LNPs is similar in wild type (WT) male cynomolgus NHPs, with little editing seen outside the liver for both LNPs. Each point represents results from an individual animal. LN denotes lymph node. Data are presented as mean values + /- standard deviation. Source data are provided as a Source Data file.

We assessed whether GalNAc-LNPs are also effective in WT NHPs in which normal LDLR activity is present. In WT NHPs, we compared GalNAc-LNPs versus standard LNPs (*N* = 3 in each treatment group), both administered a single intravenous dose of 2 mg/kg. A mean reduction in blood ANGPTL3 protein of 90% was noted for animals treated with the GalNAc-LNP versus 75% in those treated with a standard LNP (Fig. 3h). This reduction was durable when assessed six months following dosing. To determine the extent of on-target *ANGPTL3* editing in the liver and other tissues, targeted amplicon sequencing was performed in animals at time of scheduled necropsy six months following dosing. GalNAc-LNPs yielded 64% editing in WT animals, while standard LNPs yielded 58%. Assessment of 17 additional sites and tissues noted minimal editing in non-hepatic tissues with both standard and GalNAc-LNPs, less than 2% for each (Fig. 4). These data suggest that GalNAc-LNPs are effective in enabling base editing in NHPs with normal or reduced LDLR activity  and that the reduction is durable.

Liver safety monitoring noted transient increases in alanine aminotransferase (ALT) and aspartate aminotransferase (AST) for GalNAc-LNP treated NHPs, with similar results observed with standard LNPs (Supplementary Fig. 5). Maximal mean ALT values of 710 U/L were seen 48 hours after treatment with GalNAc-LNPs, while an AST maximum of 650 U/L was reached 6 hours after treatment. Both normalized to baseline values by 14 days after treatment. As in the LDLR-deficient animals, transient increases in TNF-α and MCP-1 cytokines were observed, peaking at 24 hours after dosing, with subsequent return to baseline within 7 days post-infusion. Both standard and GalNAc-LNPs were well tolerated in treated NHPs, in line with previous studies using different LNPs^11^.

WT NHPs showed little change in LDL-C (Table S4), even with substantial ANGPTL3 reduction. This is consistent with prior preclinical data in non-human primates of a monoclonal antibody targeting ANGPTL3, which did not identify a detectable difference in LDL-C even with high doses^17^. Importantly, this monoclonal antibody was shown to reduce LDL-C by approximately 50% in subsequent clinical trials in human patients^18,19^.

## Discussion

Building on prior evidence that the addition of a GalNAc-Lipid targeting ligand can facilitate hepatic delivery of siRNA and ASO therapeutics, we describe a series of experiments to optimize and validate a GalNAc-LNP capable of delivering a CRISPR base editing therapy via LDLR-independent pathways.

These results permit several conclusions. First, by systematically designing and screening various GalNAc-Lipids, we identified a configuration and LNP formulation that enables potent CRISPR base editing of the *ANGPTL3* gene. A longer PEG-spacer was more effective than a shorter one; the greater range of motion and distance from the particle afforded by a longer spacer may improve the likelihood of successful ASGPR binding. The DSG lipid anchor also outperformed other anchor structures, indicating it optimized residence time on the particle and did not interfere with endosomal escape. With a potent ligand, it was observed that a relatively low surface density of GalNAc-Lipid was required, and indeed, optimal, for enhancing ASGPR-mediated uptake and intracellular delivery. Second, the optimized GalNAc-LNP enabled efficient editing independent of LDLR. In mice studies, the observed degree of editing was nearly identical in *Ldlr*^+/+^, *Ldlr*^+/-^, and *Ldlr*^-/-^. Third, this finding in mice translated well to a NHP primate model of LDLR-deficiency, a result which had not been previously shown. In studies using LDLR-deficient and wild-type NHPs, the GalNAc-LNP achieved mean liver editing ranging from 61 to 64% and blood ANGPTL3 protein knockdown of 89 to 90%. Fourth, the observed editing was largely restricted to the target liver tissue, with minimal editing elsewhere, likely reflective of the liver-specific expression pattern of ASGPR. Fifth, stable and potent reductions in ANGPTL3 protein were observed six months following treatment with a GalNAc-LNP base editing medicine, suggesting the potential for durable or even permanent treatment effect. Sixth, the therapeutics were well-tolerated in NHPs in vivo, with transient increases in ALT, AST, and cytokines, that returned to baseline within 14 days.

These results should be interpreted within the context of several potential limitations. First, although these results indicate the potential for a GalNAc-LNP to deliver a base editing medicine for potent inactivation of the *ANGPTL3* gene, additional studies to assess for any editing at other locations in the genome (‘off-target editing’) are warranted. Second, the inactivation of *ANGPTL3* in wild-type NHP models is not expected to impact circulating LDL-C concentrations, limiting this readout of preclinical efficacy. A similar observation was noted with evinacumab, where a prior study in a dyslipidemic cynomolgus monkey model noted no change in LDL-C concentrations even after the administration of a high dose^17^. Despite this lack of change in LDL-C noted with evinacumab in non-human primates, the medicine was associated with a 47% LDL-C reduction in patients with HoFH and 50% LDL-C reduction in non-HoFH patients with increased LDL-C on maximal medical therapy^18,19^. Third, although a CRISPR base editing medicine is intended to provide potent ANGPTL3 reduction via a one-time administration, a potential advantage of use of an LNP delivery strategy, as compared to a viral vector, is lower immunogenicity that might allow for redosing^2^. Additional studies that explore the potential utility of such an approach, should it be needed, are warranted.

These precursor studies lay the foundation for the ongoing development of VERVE-201, an investigational CRISPR base editing medicine designed to target the *ANGPTL3* gene, permanently turn off hepatic protein production, and thereby durably lower LDL-C. This therapy has potential utility in addressing two patient populations with high-risk and high unmet need. The first patient population is HoFH, a rare genetic disorder afflicting approximately 1 in 250,000 individuals characterized by severe hepatic LDLR-deficiency leading to impaired removal of LDL-C from the circulation and LDL-C concentrations several times normal, and markedly accelerated atherosclerosis^13^. Most therapies in widespread clinical use for the treatment of HoFH—including statins, ezetimibe, and PCSK9 inhibitors—are dependent on the LDLR to lower circulating LDL-C concentrations, and thus have significantly less efficacy in patient with HoFH. In a recent key advance, evinacumab, a monoclonal antibody targeting ANGPTL3, was approved for use in homozygous familial hypercholesterolemia, based on reduction in LDL-C by 47% via non-LDLR dependent pathways^18^. While this result validated the potential for ANGPTL3 inactivation to enable substantial LDL-C reductions, clinical uptake has been slow, in part related to the requirement of monthly intravenous infusions. A second target patient population for VERVE-201 is refractory hypercholesterolemia, as occurs in patients with atherosclerotic cardiovascular disease who fail to achieve adequate LDL-C reduction to protect from recurrent events even after use of oral therapies and a PCSK9 inhibitor. In clinical trials of such patients treated with an siRNA targeting PCSK9, 32% of participants did not attain target LDL-C levels of <70 mg/dL. As for HoFH, prior clinical studies have validated the potential of ANGPTL3 inactivation in this population, with an observed reduction in LDL-C of 50% with the use of evinacumab^19^.

In summary, we have developed LNP delivery technology incorporating GalNAc-based ASGPR-targeting ligands and tested that technology in a NHP model of HoFH characterized by somatic LDLR-deficiency in the liver. In LDLR-deficient NHPs, administration of GalNAc-LNPs carrying an adenine base editing cargo resulted in efficient editing of the target *ANGPTL3* gene in the liver, whereas standard LNPs did not. The same GalNAc-LNPs effectively delivered an adenine base editing cargo to the livers of WT NHPs. GalNAc-LNPs provide a potent new tool for effective in vivo delivery of genome editing therapies.

## Methods

Our research complies with ethical regulations. Mouse studies were approved by the Institutional Animal Care and Use Committee of the Charles River Accelerator and Development Lab (CRADL) where the studies were performed. NHP studies were approved by the Institutional Animal Care and Use Committees of Altasciences.

### gRNA and mRNA production

ABE8.8-m mRNA was generated by an in vitro transcription reaction containing a linearized plasmid DNA template with the ABE8.8-m coding sequence and a 3’ polyadenylate sequence. The mRNA was co-transcriptionally capped and consisted of full uridine substitution with N1-methylpseudouridine.

gRNA consists of standard and chemically modified nucleotides, including ribonucleotides, 2′ O methylribonucleotides, and phosphorothioate backbone modifications. These stabilizing modifications are distributed at select positions throughout the gRNA. CRISPOR v4.98 was used to aid guide design. The dual guides utilized to generate the *LDLR*-deficient model are given in Table 1, as well as the guide targeting NHP *ANGPTL3*. We used gRNAs that were chemically synthesized under solid phase synthesis conditions by commercial suppliers with minimal end-modifications for in vitro screening and cellular screening experiments. The corresponding highly modified gRNA having the same protospacer with 2′-O-methylribosugar modifications in the design was prepared at in vivo scale (100–500 mg) for mouse and non-human primate studies.

**Table 1:** Sequences of guide RNAs utilized in this work

Target*	Protospacer (5’−3’) #	gRNA sequence (5’−3’) #
*LDLR*	gaaatgcatctcctacaagt	gsasasaugcaucuccuacaaguguuuuagagcuagaaauagcaaguuaaaauaaggcuaguccguuaucaacuugaaaaaguggcaccgagucggugcusususu
*LDLR*	gggactcatcagagccatcc	gsgsgsacucaucagagccauccguuuuagagcuagaaauagcaaguuaaaauaaggcuaguccguuaucaacuugaaaaaguggcaccgagucggugcusususu
*ANGPTL3*	aagatacctgaataactctc	asasgsauaccugaauaacucucguuuuagagcuagaaauagcaaguuaaaauaaggcuaguccguuaucaacuugaaaaaguggcaccgagucggugcusususu

# letters a, c, g, u and t indicate adenosine, cytidine, guanosine, uridine and thymidine nucleotides; (b) the lowercase letter s indicates phosphorothioate (PS) backbone.

### Preparation of standard and GalNAc-LNPs

LNPs used in the studies are listed in Tables S1 and S2. Each LNP is comprised of an ionizable amino lipid, a PEG-Lipid, cholesterol, and distearoyl-sn-glycerol-3-phosphocholine (DSPC). In addition to the standard excipients each GalNAc-LNP contains a select GalNAc-Lipid shown in Fig. 1. The GalNAc-LNPs were prepared either by mixing the ligand conjugated lipid with the lipid excipients prior to formulating the LNP or by a post-insertion process following LNP mixing. In mice, the LNPs were formulated in some experiments at an ionizable lipid:cholesterol:DSPC:PEG-lipid mol % ratio of 55:38.15:4.7:2.1. A corresponding LNP was used for NHP studies. The LNPs shown in Table S1 were constituted with the adenine base editor 8.8-m (ABE8.8) mRNA and a gRNA targeting the mouse *Angptl3* or *Pcsk9* gene. For the NHP studies, the LNPs were constituted with the adenine base editor 8.8-m (ABE8.8) mRNA and a gRNA targeting the monkey *ANGPTL3* gene (Table S2).

### LNP Analytics and characterization

LNP critical quality attributes – Z average size, polydispersity, total RNA concentration, encapsulation efficiency, and lipid content – were determined after particle formation. LNP size and polydispersity were measured using Dynamic Light Scattering via a Malvern Panalytical Zetasizer Ultra. Encapsulation efficiency was determined by fluorimetry using a Quant-iT RiboGreen RNA reagent kit (Thermo Fisher) and Triton X-100 (Sigma Aldrich), as previously described and according to manufacturer’s instructions^20^. For the evaluation of lipid composition of the LNPs, an Ion-Pairing Reverse Phase High-Performance Liquid Chromatography with evaporative light scattering detector (IP-RPLC-HPLC-ELSD) was used. The assay uses a standard curve to quantify the amino lipid, PEG-Lipid, cholesterol, DSPC, and GalNAc-Lipid. For the lectin affinity column-based analysis of GalNAc-LNP, the LNP was allowed to pass through a lectin affinity column. The flowthrough was collected and analyzed for unbound ligand-free LNPs. After washing the lectin column with loading buffer, the column was washed with PBS buffer containing D-( + )-galactose and the eluent were collected and analyzed in a similar fashion to evaluate the LNPs that bound to the lectin column.

### Animal studies

Mouse studies were approved by the Institutional Animal Care and Use Committee of the Charles River Accelerator and Development Lab (CRADL) where the studies were performed under Protocol CR-0084. Female 8-10 weeks old C57BL/6 J (Strain: 00664), *Ldlr*^+ /−^ (Strain: 002207-custom bred), and *Ldlr*^−/−^ (Strain: 002207) mice from The Jackson Laboratory were used for all mouse studies, with random assignment of mice to experimental groups. The mice were maintained on 12-h light/12-h dark cycle, with a temperature range of 65 °F to 75 °F and a humidity range of 40% to 60%. Mice were fed Prolab® Isopro® RMH 3000 5P75 and 5P76 (ScottPharma) as their diet. All animals were monitored by users at least once per week. Animals on study or with a transgenic phenotype were monitored more frequently, with the frequency determined by both the severity of the anticipated clinical signs and the expected course of disease. If animals began displaying clinical signs or demonstrating a decline in condition, monitoring frequency was increased, and a treatment plan was initiated in consultation with veterinary staff. The primary method of euthanasia was CO_2_ inhalation followed by cervical dislocation. Cessation of respiration and toe pinch reaction additionally confirmed death.

LNPs were administered to the mice via injection into the retro-orbital sinus. Five to ten days following treatment, the mice were euthanized, and liver samples were obtained on necropsy and processed with the KingFisher Flex Purification System according to the manufacturer’s instructions to isolate genomic DNA.

NHP studies were approved by the Institutional Animal Care and Use Committees of Altasciences under Protocols 138821-13 and 138821-15. Two similarly designed NHP studies were performed to confirm and extend the results, both used male cynomolgus monkeys (*Macaca fascicularis*) of Cambodian origin. The animals were 2-3 years of age and 2-3 kilograms in weight at the time of study initiation. Animals were socially housed in a temperature and humidity-controlled environment. The targeted range of temperature and relative humidity was between 18 °C and 29 °C and 30% and 70%, respectively. An automatic lighting system was set to provide a 12-hour light/dark cycle, except during designated procedures. PMI LabDiet® Fiber-Plus® Monkey Diet 5049 biscuits were provided at an appropriate daily ration and water was made available *ad libitum*. Animals were assessed for general health, appetite, and wellness at least twice daily, with cage side observations at least once daily. Animals scheduled for necropsy were sedated, weighed, and euthanized by an overdose of euthanasia solution. Separate studies have concluded that there are no discernible differences between sexes undergoing these treatments. Therefore, in order to maintain our commitment to the 3 R’s to reduce NHP use, we did not include female animals in these studies, which would have required additional animals in order to have parity between sexes in each group.

All animals were genotyped at the *ANGPTL3* editing site to ensure that any animals receiving ABE8.8/ANGPTL3 LNPs were homozygous for the protospacer DNA sequences matching the gRNA sequence; otherwise, animals were randomly assigned to various experimental groups. The animals were premedicated with 1 mg/kg dexamethasone, 0.5 mg/kg famotidine, and 5 mg/kg diphenhydramine on the day prior to LNP administration and then 30-60 minutes prior to LNP administration. The LNPs were administered via intravenous infusion into a peripheral vein over the course of 1 hour. Control animals received phosphate-buffered saline instead of LNPs under the same infusion conditions.

In both NHP experiments, WT NHPs were dosed with LNPs containing spCas9 and an *LDLR* guide RNA pair. Liver biopsies were taken at Day 19 to assess *LDLR* editing. Subsequently, at least 30 days after initial treatment, WT and newly generated somatic LDLR-deficient NHPs were injected with LNPs carrying ABE mRNA and *ANGPTL3* guide RNA. For blood chemistry samples, animals were fasted for at least 4 hours before collection via peripheral venepuncture. In both NHP studies, samples were collected on the following schedule: day –10, day –7, day –5, day 1 (6 hours after LNP infusion), day 2, day 3, day 5, day 8, and day 15. Blood samples were analysed by the study site for LDL cholesterol, HDL cholesterol, total cholesterol, triglycerides, AST, and ALT. A portion of each blood sample was used for ANGPTL3 protein measurement.

In both NHP studies, each animal underwent a liver biopsy via laparotomy on day 15 after administration of the first LNP. In one NHP study, each animal underwent euthanasia and necropsy on day 75. On necropsy, liver samples were collected by protocol. Two samples each were collected from the left, middle, right, and caudate lobes, for a total of eight samples per liver. Organ samples were processed with the KingFisher Flex Purification System (Thermo Fisher) according to the manufacturer’s instructions to isolate genomic DNA.

### NGS editing and ANGPTL3 ELISA

The DNA base editing was assessed using PCR primers specific to the targeted genomic site. Primer3 v.4.1 was used for primer design, and CRISPResso v2.0.31 and R v4.0.2 were used for data analysis and editing quantification. Briefly, PCR reactions used Accuprime High Fidelity DNA Polymerase (Thermo Fisher, #12346-094) with primers specific to the target *Angptl3* genomic site with 5’ Nextera adaptor sequences, followed by purification of the PCR amplicons with the Sequalprep Normalization Plate kit (Thermo Fisher, #A1051001). A second round of PCR with the Nextera XT Index Kit V2 Set A (Illumina, #15052163) and/or Nextera XT Index Kit V2 SetD (Illumina, #15052166), followed by purification of the PCR amplicons with the Sequalprep Normalization Plate kit, generated barcoded libraries, which were pooled and quantified using a Qubit 3.0 Fluorometer. After denaturation, dilution to 8 pM, and supplementation with 15% Phix (Illumina, #15017666), the pooled libraries underwent paired-end sequencing on an Illumina Miseq System. The NHP primer sequences are Forward: CGTCGGCAGCGTCAGATGTGTATAAGAGACAGGGGATTCGGATTTTTAAAAGTTGTC and Reverse: GTCTCGTGGGCTCGGAGATGTGTATAAGAGACAGCCCAATGCAATCCCGGAAAA with overhang sequences: Forward: TCGTCGGCAGCGTCAGATGTGTATAAGAGACAG, Reverse: GTCTCGTGGGCTCGGAGATGTGTATAAGAGACAG. The mouse primer sequences are Forward: TCGTCGGCAGCGTCAGATGTGTATAAGAGACAGGATTGCTGGCAATATCCCTGG and Reverse: GTCTCGTGGGCTCGGAGATGTGTATAAGAGACAGTGAGGAGAATGCTTGCTTGAGA.

The ANGPTL3 plasma protein levels were performed using murine or human, as appropriate, ANGPTL3-specific ELISA assays developed in our laboratory. The human ANGPTL3 ELISA kit (DANL30, R&D) was used for NHP studies, with purified cynomolgus monkey ANGPTL3 used for the calibration curve (10052-AN, R&D) and a 50-fold dilution of sample. Mouse studies utilized the mouse ANGPTL3 ELISA kit (MANL30, R&D) with a 100-fold dilution of sample^11,21^.

### Statistics

Error bars represent standard deviations (s.d.), and individual data points for each animal are displayed. The data were analyzed with GraphPad Prism v9.2.0. p values were determined via unpaired two-tailed T tests. * denotes *p* < 0.05, ** denotes *p* < 0.01, *** denotes *p* < 0.001, and **** denotes *p* < 0.0001.

### Reporting summary

Further information on research design is available in the Nature Portfolio Reporting Summary linked to this article.

## Supplementary information

Supplementary Information

Reporting Summary

Author List Changes Signed Collective Agreement

## Data Availability

All data supporting the findings described in this manuscript are available in the article, Supplementary Information, and source data file. The DNA sequencing data generated in this study have been deposited in the NCBI Sequence Read Archive database under accession code PRJNA927049 with hyperlink: https://www.ncbi.nlm.nih.gov/bioproject/PRJNA927049. The structure of the ionizable lipid and specific chemical gRNA modifications used in various experiments are not disclosed owing to proprietary considerations. Requests for this data may be directed to ‘Legal at Verve Therapeutics, Inc.’ via e-mail to legal@vervetx.com with the Subject line: “Data Request Re: A GalNAc-Lipid nanoparticle enables efficient non-LDLR dependent hepatic delivery of a CRISPR base editing therapy.” Depending on the nature of the data requests, please allow 6-8 weeks for response. These requests should include the name and full contact information of the person and institution requesting the data, the specific identification of the data being requested and the purpose of requesting the data. Data requests under agreement will be considered for purposes of reproducing the data presented herein, subject to appropriate confidentiality obligations and restrictions. Source data are provided with this paper.

## Source data

Source Data
