# Supplementary material for: GalNAc-Lipid nanoparticles enable non-LDLR dependent hepatic delivery of a CRISPR base editing therapy
Source: Nat Commun. 2023 May 15;14:2776. doi: 10.1038/s41467-023-37465-1 (PMC10185539; doi:10.1038/s41467-023-37465-1)
Supplement: Supplementary file 1 — Supplementary Information [file 41467_2023_37465_MOESM1_ESM.pdf]

Supplemental Information

Standard LNPs and GalNAc-LNPs

Table S1: LNP characterization and dosing data for Figures 1, 2, S1, S3, and S4, which were dosed into mice via retro-orbital injection at 10 mL/kg.

LNP	Mouse Targets[a]	GalNAc-Lipid[b] Compound ID	mol %	Method of incorporation	Average LNP size (nm)	PDI	RNA entrapment (%)	Dose (mg/kg)	Figure
1	*Angptl3*	GL6	0.05	c	98.61	0.013	94.91	0.1	1d
2	*Angptl3*	GL3	0.05	c	100.4	0.023	94.27	0.1	1d
3	*Pcsk9*	GL6	0.5	d	79.3	0.072	94.7	0.25	1e
4	*Pcsk9*	GL3	0.5	d	74.1	0.036	96	0.25	1e
5	*Angptl3*	GL5	0.05	c	100.6	0.029	92.48	0.3	1f, S1(a)
6	*Angptl3*	GL6	0.05	c	100.4	0.034	93.59	0.3	1f, 1g, S1
7	*Angptl3*	GL7	0.05	c	98.78	0.032	92.86	0.3	1g, S1(b)
8	*Angptl3*	GL9	0.05	c	100.9	0.002	92.65	0.3	1g, S1(b)
9	*Angptl3*	GL6	0	N/A	106.6	0.01	94.85	0.1	2b
10	*Angptl3*	GL6	0.01	c	98.35	0.016	95.01	0.1	2b
11	*Angptl3*	GL6	0.05	c	98.61	0.013	94.91	0.1	2b
12	*Angptl3*	GL6	0.25	c	95.98	0.010	95.84	0.1	2b
13	*Angptl3*	GL6	0.5	c	89.63	0.025	96.15	0.1	2b
14	*Angptl3*	GL6	1	c	98.35	0.016	95.01	0.1	2b
15	*Angptl3*	GL6	0	N/A	68.09	0.002	98.66	0.25	2c, S4(a)
16	*Angptl3*	GL6	0.05	c	66.73	0.018	98.36	0.25	2c, S4(a)
17	*Angptl3*	GL6	0.05	c	77.24	0.028	99.2	0.1, 0.25, 0.5	2d, S4(b)
18	*Angptl3*	GL6	0.05	d	120.6	0.013	94.91	0.1	S3
19	*Angptl3*	GL6	0.05	c	98.61	0.011	94.27	0.1	S3

[a] The mouse *Angptl3* and *Pcsk9* gRNAs used in these studies were selected from Chadwick *et al.* WO2021178725.
[b] The GalNAc-Lipids GL3 and GL6 were synthesized and characterized as described in Rajeev *et al.* WO2021178725.
[c] GalNAc-Lipid is premixed with other LNP excipients prior to in-line mixing with RNA to form the GalNAc-LNPs as described in Rajeev *et al.* WO2021178725.
[d] GalNAc-Lipid was post-inserted into the LNP to obtain the desired GalNAc-LNP as described in Rajeev *et al.,* WO2021178725.
N/A: not applicable

Table S2: LNPs characterization and dosing data for generating the data shown in Figs. 3 and 4.

LNP	NHP Target[a]	mol % GalNAc-Lipid GL6[b]	Average LNP size (nm)	PDI	RNA entrapment (%)	Dose (mg/kg)	Figure
1	*ANGPTL3*	0	74.97	0.057	92.6	2	3e, 3f
2	*ANGPTL3*	0	72.73	0.032	94.3	2	3h, 4
3	*ANGPTL3*	0.05	72.93	0.083	93.6	2	3e, 3f
4	*ANGPTL3*	0.05	71.97	0.042	93.3	2	3e, 3g, 3h, 4

[a] The NHP *ANGPTL3* gRNA used in these studies was selected from Chadwick *et al.* WO2021178725.
[b] GalNAc-Lipid is premixed with other LNP excipients prior to in-line mixing with RNA to form the desired GalNAc-LNPs as described in Rajeev *et al.,* WO2021178725.

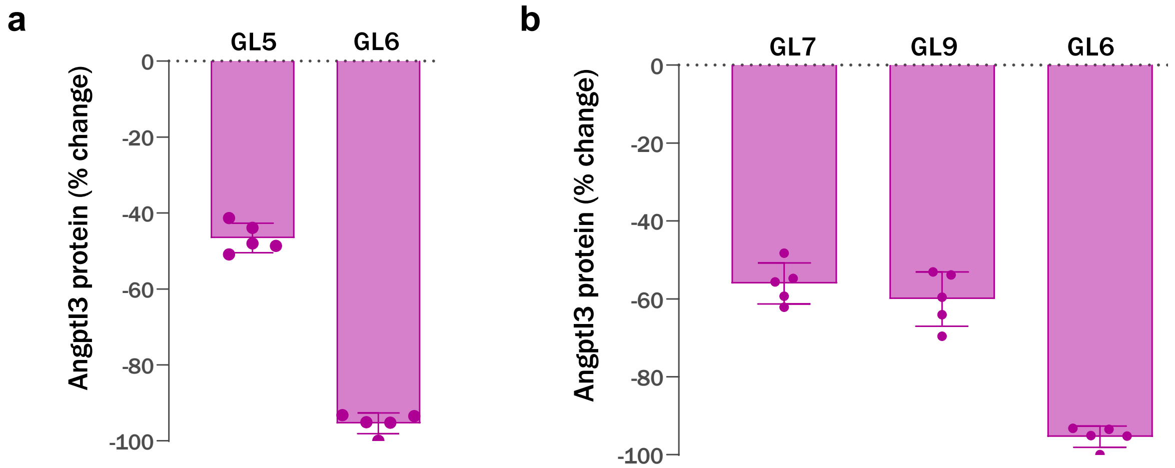

Figure S1: Structure-based evaluation of trivalent GalNAc-ligands with different spacers and anchors in *Ldlr*$^{-/-}$ mice at a dose of 0.3 mg/kg, as seen in Fig. 1. (a) GalNAc-LNPs formulated with the longer PEG spacer of GL6 (Table S1, entry 6) achieved a greater reduction in Angptl3 protein than the GalNAc-LNPs with the shorter PEG spacer of GL5 (Table S1 entry 5). (b) Modulation of the lipid tail hydrophobicity in GL7 and GL9 (Table S1, entries 7 and 8) was unable to improve the Angptl3 protein reduction of GalNAc-LNPs in *Ldlr*$^{-/-}$ mice compared to GL6 (Table S1, entry 6). Data are presented as mean values +/- standard deviation. Source data are provided as a Source Data file. (N = 5)

a)

Flowthrough

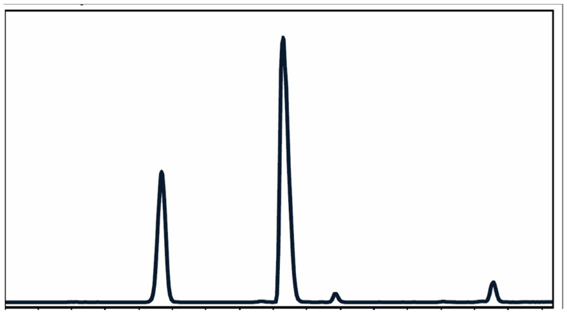

LNPs without GalNAc-Lipid do not bind to lectin affinity column

b)

Elution

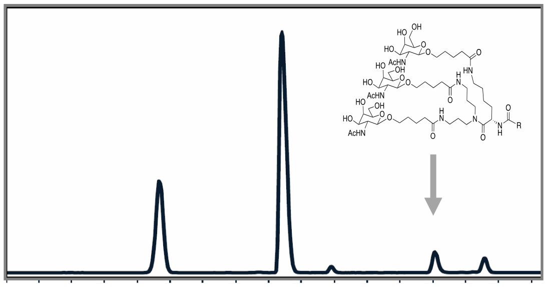

LNPs with GalNAc-Lipid GL6 bind and elute from the lectin affinity column

Figure S2: Confirmation of GalNAc-Lipid incorporation into the lipid nanoparticle (LNP). We generated GalNAc-LNPs with 0.5 mol % GL6 via a post-addition method of GalNAc-Lipid incorporation. These LNPs were then passed through a lectin affinity column, with PBS flowed into the column afterwards. LNPs containing GalNAc-Lipid would be expected to bind to the lectin column. Fractions of the flowthrough were then collected. If all LNPs contained GalNAc-Lipid, no LNPs would be expected in this flowthrough. The lectin column was then washed with PBS containing D-(+)-galactose and the column eluent was collected. Galactose is expected to displace GalNAc-LNPs from the column and now allow them to be eluted in the flowthrough. (a) Fractions collected from the flowthrough and rinse fractions in PBS when analyzed by IP-RPLC-HPLC-ELSD showed the presence of all 4 lipids in their expected molar composition. This indicates that a population of the post-addition manufactured LNPs do not actually contain GalNAc-Lipid at all, as they were unable to bind to the lectin column and were flushed out by the PBS. (b) Fractions collected from the elution with PBS containing D-(+)-Galactose showed GalNAc-Lipid in addition to the other four LNP excipients in the expected ratio. These data

indicate certain population, but not all, of the LNPs generated via the post-addition method contained GalNAc-Lipid, and so were displaced as expected by the galactose. The non-homogenous distribution of GalNAc-Lipid with post-addition incorporation motivated the development of in-lipid mixing formulations.

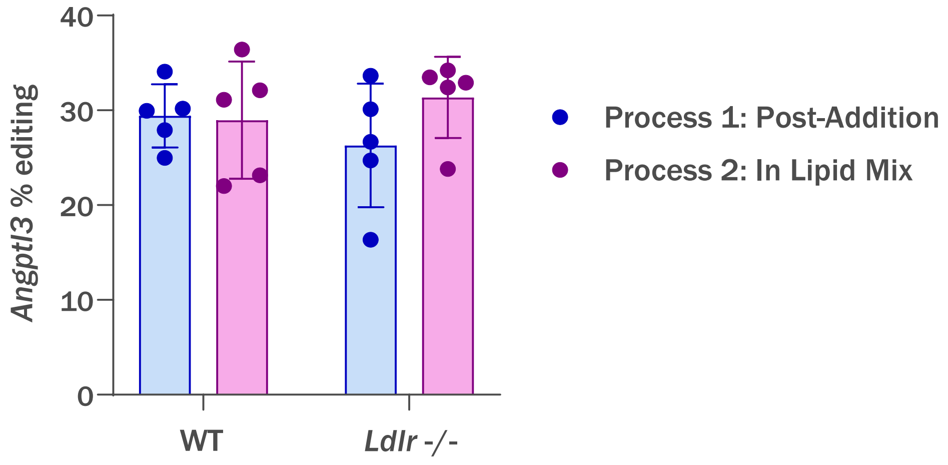

Figure S3: GalNAc-LNPs were formulated with 0.05 mol % GalNAc-Lipid GL6 added via post-addition or in-lipid mixing methods (Table S1, entries 18 and 19). GalNAc-LNPs prepared by both methods produced nearly identical editing in wild type (WT) and *Ldlr* –/– mice at a 0.1 mg/kg dose. Data are presented as mean values +/- standard deviation. Source data are provided as a Source Data file. (N = 5 biologically independent mice.)

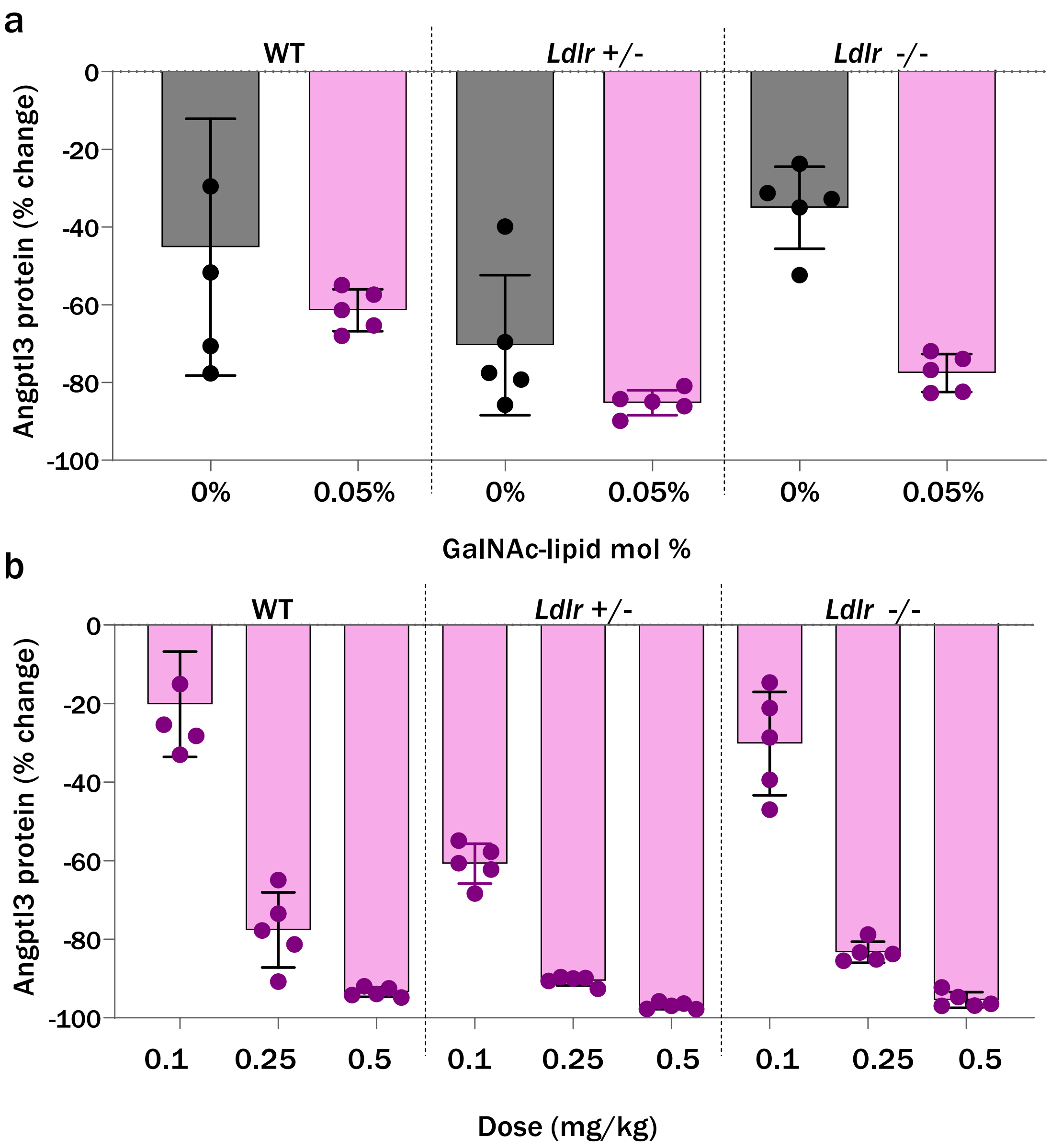

Figure S4: Angptl3 protein reductions corresponding to *Angptl3* editing findings in Figs. 2c and 2d. (a) LNPs formulated with and without 0.05 mol % GL6 were administered to WT, *Ldlr*$^{+/-}$, and *Ldlr*$^{-/-}$ mice at 0.25 mg/kg. The inclusion of GalNAc-Lipid GL6 rescued activity in *Ldlr*$^{-/-}$ mice and maintained activity in WT and *Ldlr*$^{+/-}$ (Table S1, entries 15 and 16). (b) 0.05 mol % GL6 GalNAc-LNPs were administered at the stated doses to WT, *Ldlr*$^{+/-}$, and *Ldlr*$^{-/-}$ mice. Dose-dependent Angptl3 protein reduction was achieved in all three animal models with the GalNAc-LNP evaluated (Table S1, entry 17). Data are presented as mean values +/- standard deviation.

Source data are provided as a Source Data file. (N = 5 biologically independent mice, except for 0% GalNAc-lipid in WT, which was N = 4 biologically independent mice.)

Animal	Percentage of deletions of length 31-40 bp	Total percentage of insertions or deletions	Percentage of deletions of length 31-40 bp with respect to total indels
1	56.38	64.28	87.7
2	68.83	74.62	92.2
3	60.82	67.29	90.4
4	63.09	68.92	91.5
5	67.74	73.04	92.7
6	62.17	68.23	91.1
7	55.8	61.32	91.0
8	65.71	71.88	91.4
9	51.39	58.23	88.3
10	67.86	73	93.0

Table S3: Indel creation in LDLR deficient NHP model using dual Cas9 guide-loaded LNPs.

	LDL-C Absolute values (mg/dL)					
Days Post Infusion	Standard LNPs			GalNAc-LNPs		
0	35.7	39.3	69.3	38.7	59	76.7
7	36	31	50	28	53	61
14	32	35	53	23	52	64
21	31	45	62	33	60	73
28	30	37	51	29	58	71
42	32	42	64	41	67	82
56	32	38	57	38	59	75
70	39	41	64	42	66	85
84	36	43	55	30	57	68
90	39	40	57	30	51	78

Table S4: Absolute LDL-C values (mg/dL) for WT NHPs treated with standard and GalNAc-LNPs at a 2 mg/kg dose, as depicted in Figure 3.

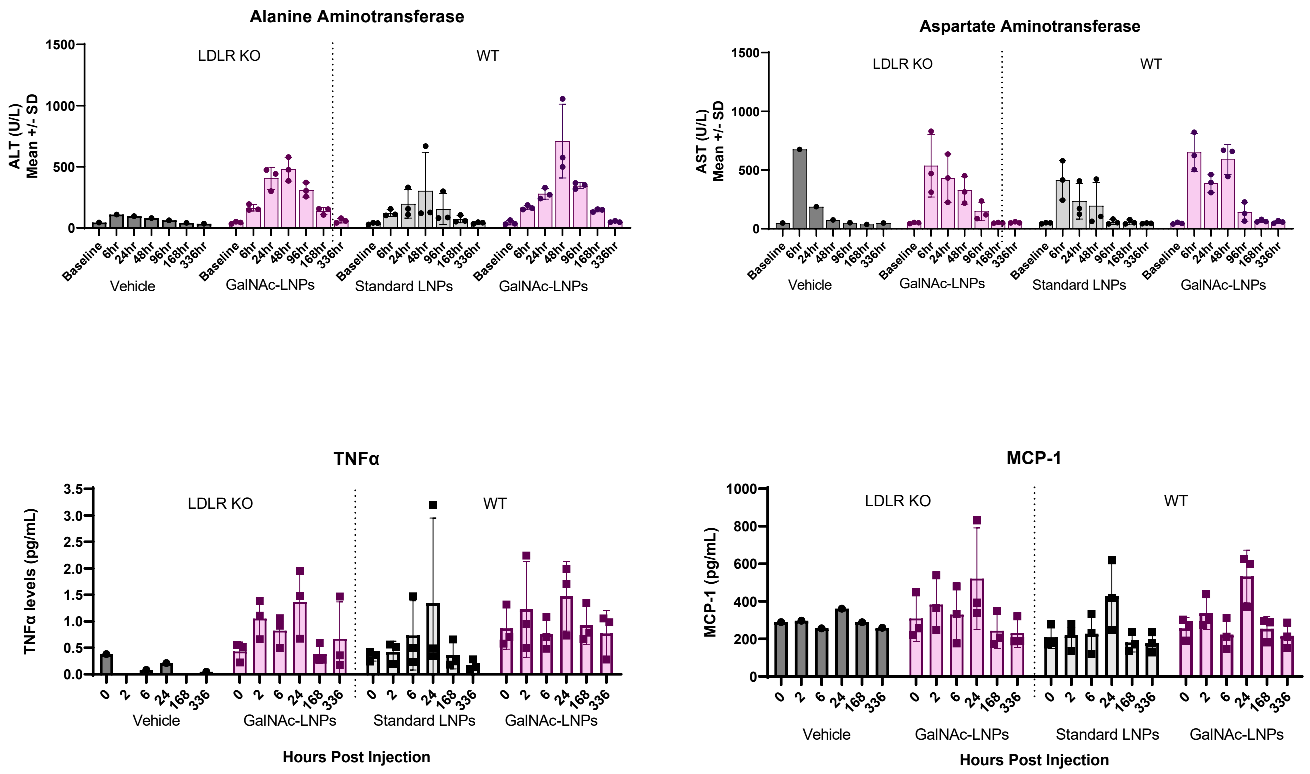

Figure S5: Liver toxicity and innate immune cytokine increases for low density lipoprotein receptor (*LDLR*) deficient and wild type (WT) NHPs treated with standard lipid nanoparticles (LNPs) and GalNAc-LNPs are transient and return to baseline. (a) alanine aminotransferase (ALT), (b) aspartate aminotransferase (AST), (c) tumor necrosis factor α (TNFα), and (d) monocyte chemoattractant protein 1 (MCP-1) measured out to 14 days following 2 mg/kg doses of standard or GalNAc-LNPs. Data are presented as mean values +/- standard deviation. Source data are provided as a Source Data file. (N = 3 biologically independent non-human primates.)
