# Supplementary material for: GalNAc-Lipid nanoparticles enable non-LDLR dependent hepatic delivery of a CRISPR base editing therapy
Source: Nat Commun. 2023 May 15;14:2776. doi: 10.1038/s41467-023-37465-1 (PMC10185539; doi:10.1038/s41467-023-37465-1)
Supplement: Supplementary file 2 — Reporting Summary [file 41467_2023_37465_MOESM2_ESM.pdf]

nature portfolio

Corresponding author(s): Andrew Bellinger

Last updated by author(s): Mar 3, 2023

Reporting Summary

Nature Portfolio wishes to improve the reproducibility of the work that we publish. This form provides structure for consistency and transparency in reporting. For further information on Nature Portfolio policies, see our Editorial Policies and the Editorial Policy Checklist.

Please do not complete any field with "not applicable" or n/a. Refer to the help text for what text to use if an item is not relevant to your study.
For final submission: please carefully check your responses for accuracy; you will not be able to make changes later.

Statistics

For all statistical analyses, confirm that the following items are present in the figure legend, table legend, main text, or Methods section.

n/a	Confirmed	
☐	☒	The exact sample size (*n*) for each experimental group/condition, given as a discrete number and unit of measurement
☐	☒	A statement on whether measurements were taken from distinct samples or whether the same sample was measured repeatedly
☐	☒	The statistical test(s) used AND whether they are one- or two-sided<br>*Only common tests should be described solely by name; describe more complex techniques in the Methods section.*
☒	☐	A description of all covariates tested
☒	☐	A description of any assumptions or corrections, such as tests of normality and adjustment for multiple comparisons
☐	☒	A full description of the statistical parameters including central tendency (e.g. means) or other basic estimates (e.g. regression coefficient) AND variation (e.g. standard deviation) or associated estimates of uncertainty (e.g. confidence intervals)
☐	☒	For null hypothesis testing, the test statistic (e.g. *F*, *t*, *r*) with confidence intervals, effect sizes, degrees of freedom and *P* value noted<br>*Give P values as exact values whenever suitable.*
☒	☐	For Bayesian analysis, information on the choice of priors and Markov chain Monte Carlo settings
☒	☐	For hierarchical and complex designs, identification of the appropriate level for tests and full reporting of outcomes
☒	☐	Estimates of effect sizes (e.g. Cohen's *d*, Pearson's *r*), indicating how they were calculated

Our web collection on statistics for biologists contains articles on many of the points above.

Software and code

Policy information about availability of computer code

Data collection	*Provide a description of all commercial, open source and custom code used to collect the data in this study, specifying the version used OR state that no software was used.*
Data analysis	CRISPResso2 v2.0.31, R v4.0.2, GraphPad Prism v9.2.0, CRISPOR v4.98, Primer3 v.4.1

For manuscripts utilizing custom algorithms or software that are central to the research but not yet described in published literature, software must be made available to editors and reviewers. We strongly encourage code deposition in a community repository (e.g. GitHub). See the Nature Portfolio guidelines for submitting code & software for further information.

Data

Policy information about availability of data

All manuscripts must include a data availability statement. This statement should provide the following information, where applicable:

- Accession codes, unique identifiers, or web links for publicly available datasets
- A description of any restrictions on data availability
- For clinical datasets or third party data, please ensure that the statement adheres to our policy

All data supporting the findings described in this manuscript are available in the article, Supplementary Information, and source data file. The DNA sequencing data generated in this study have been deposited in the NCBI Sequence Read Archive database under accession code PRJNA927049 with hyperlink: https://www.ncbi.nlm.nih.gov/bioproject/PRJNA927049. The structure of the ionizable lipid and specific chemical gRNA modifications used in various experiments are not

disclosed owing to proprietary considerations. Requests for this data may be directed to 'Legal at Verve Therapeutics, Inc.' via e-mail to legal@vervetx.com with the Subject line: "Data Request Re: A GalNAc-lipid nanoparticle enables efficient non-LDLR dependent hepatic delivery of a CRISPR base editing therapy." Depending on the nature of the data requests, please allow 6-8 weeks for response. These requests should include the name and full contact information of the person and institution requesting the data, the specific identification of the data being requested and the purpose of requesting the data. Data requests under agreement will be considered for purposes of reproducing the data presented herein, subject to appropriate confidentiality obligations and restrictions. Source data are provided with this paper.

Human research participants

Policy information about studies involving human research participants and Sex and Gender in Research.

Reporting on sex and gender	No human research participants were involved in this work.
Population characteristics	*Describe the covariate-relevant population characteristics of the human research participants (e.g. age, genotypic information, past and current diagnosis and treatment categories). If you filled out the behavioural & social sciences study design questions and have nothing to add here, write "See above."*
Recruitment	*Describe how participants were recruited. Outline any potential self-selection bias or other biases that may be present and how these are likely to impact results.*
Ethics oversight	*Identify the organization(s) that approved the study protocol.*

Note that full information on the approval of the study protocol must also be provided in the manuscript.

Field-specific reporting

Please select the one below that is the best fit for your research. If you are not sure, read the appropriate sections before making your selection.

☒ Life sciences ☐ Behavioural & social sciences ☐ Ecological, evolutionary & environmental sciences

For a reference copy of the document with all sections, see nature.com/documents/nr-reporting-summary-flat.pdf

Life sciences study design

All studies must disclose on these points even when the disclosure is negative.

Sample size	Sample sizes were determined based on literature precedent as well as ethical consideration (using the minimum number of animals needed for experimentation). The total number of animals is the minimum needed to properly characterize responses related to Test Article administration and, thus, to meet experimental objectives. The studies presented herein were non-regulated pharmacology studies, and so were not powered to provide statistical significance. The number of animals is the minimum that we require, based on historical data, to generate reliable data while maintaining our commitment to the 3R's.
Data exclusions	No data was excluded.
Replication	The LDLR deficient NHP data was repeated twice, as it is derived from two separate NHP studies wherein the LDLR deficient model was created and the NHPs were dosed with GalNAc-LNPs. Both experiments were successful and yielded similar editing results. Mouse data was successfully repeated at least once with a comparable LNP.
Randomization	Randomization was used when feasible for mouse and NHP studies. An important exception was exclusion of a non-human primate(s) from a treatment group if the genotype at the genome editing site did not match the treatment (e.g. protospacer DNA sequence.)
Blinding	The investigators were not blinded during collection or analysis. NHP studies were performed at contract research organizations. Blinding of the animal care staff and technicians would have been difficult to achieve due to the practice of social housing. Animals receiving the same treatment are housed with social access to one another. The staff would also not be blinded to the assigned group as the study ID of the animal contains the group number. Also, it is at least assumed that group 1 may be a vehicle control since it is dosed first to avoid any potential cross-contamination. Finally, it would have been nearly impossible to blind Verve investigators due to the nature of the study design where a subset of animals was first treated to induce the LDLR knock down, and those animals were then stratified into new treatment groups along with additional wild-type animals. The treatment paradigm had to be known to Verve investigators in order to properly assign animals.

Reporting for specific materials, systems and methods

We require information from authors about some types of materials, experimental systems and methods used in many studies. Here, indicate whether each material, system or method listed is relevant to your study. If you are not sure if a list item applies to your research, read the appropriate section before selecting a response.

Materials & experimental systems

n/a	Involved in the study
☐	☒ Antibodies
☒	☐ Eukaryotic cell lines
☒	☐ Palaeontology and archaeology
☐	☒ Animals and other organisms
☒	☐ Clinical data
☒	☐ Dual use research of concern

Methods

n/a	Involved in the study
☒	☐ ChIP-seq
☒	☐ Flow cytometry
☒	☐ MRI-based neuroimaging

Antibodies

Antibodies used	The ANGPTL3 plasma protein levels were performed using murine or human, as appropriate, ANGPTL3-specific ELISA assays developed in our laboratory. The human ANGPTL3 ELISA kit (DANL30, R&D) was used for NHP studies, with purified cynomolgus monkey ANGPTL3 used for the calibration curve (10052-AN, R&D) and a 50-fold dilution of sample. Mouse studies utilized the mouse ANGPTL3 ELISA kit (MANL30, R&D) with a 100-fold dilution of sample.
Validation	The vendor provides precision/recovery/linearity/sensitivity of the ELISA kit, as well as kit directions.

Animals and other research organisms

Policy information about studies involving animals; ARRIVE guidelines recommended for reporting animal research, and Sex and Gender in Research

Laboratory animals	NHP studies used male cynomolgus monkeys (Macaca fascicularis) of Cambodian origin. The animals were 2-3 years of age and 2-3 kilograms in weight at the time of study initiation. They were housed at Altasciences. Female 8-10 weeks old C57BL/6J, Ldlr +/–, and Ldlr –/– mice from The Jackson Laboratory were used for the mouse studies. The mice were maintained on 12-h light/12-h dark cycle, with a temperature range of 65 °F to 75 °F and a humidity range of 40% to 60%.
Wild animals	No wild animals were used in this study.
Reporting on sex	Separate studies have concluded that there are no discernible differences between sexes undergoing these treatments. Therefore, in order to maintain our commitment to the 3R’s to reduce NHP use, we did not include female animals in these studies, which would have required additional animals in order to have parity between sexes in each group.
Field-collected samples	This study did not involve samples collected from the field.
Ethics oversight	Mouse studies were approved by the Institutional Animal Care and Use Committee of the Charles River Accelerator and Development Lab (CRADL) where the studies were performed under Protocol CR-0084. NHP studies were approved by the Institutional Animal Care and Use Committees of Altasciences under Protocols 138821-13 and 138821-15.

Note that full information on the approval of the study protocol must also be provided in the manuscript.

This checklist template is licensed under a Creative Commons Attribution 4.0 International License, which permits use, sharing, adaptation, distribution and reproduction in any medium or format, as long as you give appropriate credit to the original author(s) and the source, provide a link to the Creative Commons license, and indicate if changes were made. The images or other third party material in this article are included in the article's Creative Commons license, unless indicated otherwise in a credit line to the material. If material is not included in the article's Creative Commons license and your intended use is not permitted by statutory regulation or exceeds the permitted use, you will need to obtain permission directly from the copyright holder. To view a copy of this license, visit http://creativecommons.org/licenses/by/4.0/
