# Supplementary material for: GalNAc-Lipid nanoparticles enable non-LDLR dependent hepatic delivery of a CRISPR base editing therapy
Source: Nat Commun. 2023 May 15;14:2776. doi: 10.1038/s41467-023-37465-1 (PMC10185539; doi:10.1038/s41467-023-37465-1)
Supplement: Supplementary file 3 — Author List Changes Signed Collective Agreement [file 41467_2023_37465_MOESM3_ESM.pdf]

Lisa Kasiewicz

From: Lisa Kasiewicz
Sent: Thursday, March 2, 2023 12:43 PM
To: Lisa Kasiewicz
Subject: RE: REPLY REQUESTED: Author change for GalNAc Manuscript NCOMMS-22-08508C

I also give my permission.

"As a coauthor of this manuscript, I agree with the addition of Dr. Yuri Matsumoto to the author list as outlined below."

Updated byline is below.

GalNAc-Lipid nanoparticles enable non-LDLR dependent hepatic delivery of a CRISPR base editing therapy

Lisa N. Kasiewicz[1], Souvik Biswas[1], Aaron Beach[1], Huilan Ren[1], Chaitali Dutta[1], Anne Marie Mazzola[1], Ellen Rohde[1], Alexandra Chadwick[1], Christopher Cheng[1], Sara P. Garcia[1],
Sowmya Iyer[1], **Yuri Matsumoto**[1], Amit V. Khera[1], Kiran Musunuru[2], Sekar Kathiresan[1], Padma Malyala[1], Kallanthottathil G. Rajeev[1], and Andrew M. Bellinger[1*]

From: Lisa Kasiewicz
Sent: Wednesday, March 1, 2023 3:09 PM
To: Souvik Biswas <sbiswas@vervetx.com>; Aaron Beach <abeach@vervetx.com>; Huilan Ren <hren@vervetx.com>; Chaitali Dutta <cdutta@vervetx.com>; Anne Marie Mazzola <ammazzola@vervetx.com>; Ellen Rohde <erohde@vervetx.com>; Alexandra Chadwick <achadwick@vervetx.com>; Christopher Cheng <ccheng@vervetx.com>; Sara Garcia <sgarcia@vervetx.com>; Amit Khera <akhera@vervetx.com>; Kiran Musunuru <kmusunuru@vervetx.com>; Sekar Kathiresan <skathiresan@vervetx.com>; Padma Malyala <pmalyala@vervetx.com>; Kallanthottathil Rajeev <krajeev@vervetx.com>; Andrew Bellinger <abellinger@vervetx.com>; sowmyai@bu.edu; Yuri Matsumoto <ymatsumoto@vervetx.com>
Subject: REPLY REQUESTED: Author change for GalNAc Manuscript NCOMMS-22-08508C
Importance: High

Dear Team:

Nature Communications requires all-author approval when the author list is changed. Dr. Yuri Matsumoto was mistakenly left off of the authors list and will thus be added as a correction. I will be collating these responses and providing them to *Nature Communications*.

Can you please each reply to this e-mail with the statement:
"As a coauthor of this manuscript, I agree with the addition of Dr. Yuri Matsumoto to the author list as outlined below."

Updated byline is below.

GalNAc-Lipid nanoparticles enable non-LDLR dependent hepatic delivery of a CRISPR base editing therapy

Lisa N. Kasiewicz[1], Souvik Biswas[1], Aaron Beach[1], Huilan Ren[1], Chaitali Dutta[1], Anne Marie Mazzola[1], Ellen Rohde[1], Alexandra Chadwick[1], Christopher Cheng[1], Sara P. Garcia[1],

Sowmya Iyer[1], **Yuri Matsumoto**[1], Amit V. Khera[1], Kiran Musunuru[2], Sekar Kathiresan[1], Padma Malyala[1], Kallanthottathil G. Rajeev[1], and Andrew M. Bellinger[1*]

Thank you very much for your assistance,
Lisa

Lisa Kasiewicz, PhD
Associate Principal Scientist, Formulation

O: (617) 603-0070 **M:** (203) 673-9730
E: lkasiewicz@vervetx.com
A: 201 Brookline Avenue, Suite 601| Boston, MA 02215
www.vervetx.com

CONFIDENTIALITY NOTICE: The information contained in this communication from the sender is confidential. It is intended solely for use by the recipient and others authorized to receive it. If you are not the recipient, you are hereby notified that any disclosure, copying, distribution or taking action in relation of the contents of this information is strictly prohibited and may be unlawful.

Lisa Kasiewicz

From: Souvik Biswas
Sent: Wednesday, March 1, 2023 4:14 PM
To: Lisa Kasiewicz; Aaron Beach; Huilan Ren; Chaitali Dutta; Anne Marie Mazzola; Ellen Rohde; Alexandra Chadwick; Christopher Cheng; Sara Garcia; Amit Khera; Kiran Musunuru; Sekar Kathiresan; Padma Malyala; Kallanthottathil Rajeev; Andrew Bellinger; sowmyai@bu.edu; Yuri Matsumoto
Subject: RE: REPLY REQUESTED: Author change for GalNAc Manuscript NCOMMS-22-08508C

As a coauthor of this manuscript, I agree with the addition of Dr. Yuri Matsumoto to the author list as outlined below.

GalNAc-Lipid nanoparticles enable non-LDLR dependent hepatic delivery of a CRISPR base editing therapy

Regards,
Souvik

From: Lisa Kasiewicz <lkasiewicz@vervetx.com>
Sent: Wednesday, March 1, 2023 3:09 PM
To: Souvik Biswas <sbiswas@vervetx.com>; Aaron Beach <abeach@vervetx.com>; Huilan Ren <hren@vervetx.com>; Chaitali Dutta <cdutta@vervetx.com>; Anne Marie Mazzola <ammazzola@vervetx.com>; Ellen Rohde <erohde@vervetx.com>; Alexandra Chadwick <achadwick@vervetx.com>; Christopher Cheng <ccheng@vervetx.com>; Sara Garcia <sgarcia@vervetx.com>; Amit Khera <akhera@vervetx.com>; Kiran Musunuru <kmusunuru@vervetx.com>; Sekar Kathiresan <skathiresan@vervetx.com>; Padma Malyala <pmalyala@vervetx.com>; Kallanthottathil Rajeev <krajeev@vervetx.com>; Andrew Bellinger <abellinger@vervetx.com>; sowmyai@bu.edu; Yuri Matsumoto <ymatsumoto@vervetx.com>
Subject: REPLY REQUESTED: Author change for GalNAc Manuscript NCOMMS-22-08508C
Importance: High

Dear Team:

Nature Communications requires all-author approval when the author list is changed. Dr. Yuri Matsumoto was mistakenly left off of the authors list and will thus be added as a correction. I will be collating these responses and providing them to *Nature Communications*.

Can you please each reply to this e-mail with the statement:

> **"As a coauthor of this manuscript, I agree with the addition of Dr. Yuri Matsumoto to the author list as outlined below."**

Updated byline is below.

GalNAc-Lipid nanoparticles enable non-LDLR dependent hepatic delivery of a CRISPR base editing therapy

Lisa N. Kasiewicz[1], Souvik Biswas[1], Aaron Beach[1], Huilan Ren[1], Chaitali Dutta[1], Anne Marie Mazzola[1], Ellen Rohde[1], Alexandra Chadwick[1], Christopher Cheng[1], Sara P. Garcia[1],
Sowmya Iyer[1], **Yuri Matsumoto**[1], Amit V. Khera[1], Kiran Musunuru[2], Sekar Kathiresan[1], Padma Malyala[1], Kallanthottathil G. Rajeev[1], and Andrew M. Bellinger[1*]

Lisa Kasiewicz

From: Aaron Beach
Sent: Wednesday, March 1, 2023 4:42 PM
To: Lisa Kasiewicz; Souvik Biswas; Huilan Ren; Chaitali Dutta; Anne Marie Mazzola; Ellen Rohde; Alexandra Chadwick; Christopher Cheng; Sara Garcia; Amit Khera; Kiran Musunuru; Sekar Kathiresan; Padma Malyala; Kallanthottathil Rajeev; Andrew Bellinger; sowmyai@bu.edu; Yuri Matsumoto
Subject: RE: REPLY REQUESTED: Author change for GalNAc Manuscript NCOMMS-22-08508C

As a coauthor of this manuscript, I agree with the addition of Dr. Yuri Matsumoto to the author list as outlined below.

GalNAc-Lipid nanoparticles enable non-LDLR dependent hepatic delivery of a CRISPR base editing therapy

Aaron Beach (he/his)
Senior Director – Internal Quality Control

O: (937) 287-6157 **M:** (937) 287-6157
E: abeach@vervetx.com
A: 201 Brookline Avenue, Suite 601, Boston, MA 02215
www.vervetx.com

CONFIDENTIALITY NOTICE: The information contained in this communication from the sender is confidential. It is intended solely for use by the recipient and others authorized to receive it. If you are not the recipient, you are hereby notified that any disclosure, copying, distribution or taking action in relation of the contents of this information is strictly prohibited and may be unlawful.

From: Lisa Kasiewicz <lkasiewicz@vervetx.com>
Sent: Wednesday, March 1, 2023 3:09 PM
To: Souvik Biswas <sbiswas@vervetx.com>; Aaron Beach <abeach@vervetx.com>; Huilan Ren <hren@vervetx.com>; Chaitali Dutta <cdutta@vervetx.com>; Anne Marie Mazzola <ammazzola@vervetx.com>; Ellen Rohde <erohde@vervetx.com>; Alexandra Chadwick <achadwick@vervetx.com>; Christopher Cheng <ccheng@vervetx.com>; Sara Garcia <sgarcia@vervetx.com>; Amit Khera <akhera@vervetx.com>; Kiran Musunuru <kmusunuru@vervetx.com>; Sekar Kathiresan <skathiresan@vervetx.com>; Padma Malyala <pmalyala@vervetx.com>; Kallanthottathil Rajeev <krajeev@vervetx.com>; Andrew Bellinger <abellinger@vervetx.com>; sowmyai@bu.edu; Yuri Matsumoto <ymatsumoto@vervetx.com>
Subject: REPLY REQUESTED: Author change for GalNAc Manuscript NCOMMS-22-08508C
Importance: High

Dear Team:

Nature Communications requires all-author approval when the author list is changed. Dr. Yuri Matsumoto was mistakenly left off of the authors list and will thus be added as a correction. I will be collating these responses and providing them to *Nature Communications*.

Can you please each reply to this e-mail with the statement:

> **"As a coauthor of this manuscript, I agree with the addition of Dr. Yuri Matsumoto to the author list as outlined below."**

Updated byline is below.

GalNAc-Lipid nanoparticles enable non-LDLR dependent hepatic delivery of a CRISPR base editing therapy

Lisa N. Kasiewicz[1], Souvik Biswas[1], Aaron Beach[1], Huilan Ren[1], Chaitali Dutta[1], Anne Marie Mazzola[1], Ellen Rohde[1], Alexandra Chadwick[1], Christopher Cheng[1], Sara P. Garcia[1],
Sowmya Iyer[1], **Yuri Matsumoto[1]**, Amit V. Khera[1], Kiran Musunuru[2], Sekar Kathiresan[1], Padma Malyala[1], Kallanthottathil G. Rajeev[1], and Andrew M. Bellinger[1]*

Thank you very much for your assistance,
Lisa

Lisa Kasiewicz, PhD
Associate Principal Scientist, Formulation

verve THERAPEUTICS

O: (617) 603-0070 **M:** (203) 673-9730
E: lkasiewicz@vervetx.com
A: 201 Brookline Avenue, Suite 601| Boston, MA 02215
www.vervetx.com

CONFIDENTIALITY NOTICE: The information contained in this communication from the sender is confidential. It is intended solely for use by the recipient and others authorized to receive it. If you are not the recipient, you are hereby notified that any disclosure, copying, distribution or taking action in relation of the contents of this information is strictly prohibited and may be unlawful.

Lisa Kasiewicz

From: Huilan Ren
Sent: Wednesday, March 1, 2023 3:11 PM
To: Lisa Kasiewicz
Subject: RE: REPLY REQUESTED: Author change for GalNAc Manuscript NCOMMS-22-08508C

Hi Lisa,

As a coauthor of this manuscript, I agree with the addition of Dr. Yuri Matsumoto to the author list as outlined below.

Best,
Huilan

From: Lisa Kasiewicz <lkasiewicz@vervetx.com>
Sent: Wednesday, March 1, 2023 3:09 PM
To: Souvik Biswas <sbiswas@vervetx.com>; Aaron Beach <abeach@vervetx.com>; Huilan Ren <hren@vervetx.com>; Chaitali Dutta <cdutta@vervetx.com>; Anne Marie Mazzola <ammazzola@vervetx.com>; Ellen Rohde <erohde@vervetx.com>; Alexandra Chadwick <achadwick@vervetx.com>; Christopher Cheng <ccheng@vervetx.com>; Sara Garcia <sgarcia@vervetx.com>; Amit Khera <akhera@vervetx.com>; Kiran Musunuru <kmusunuru@vervetx.com>; Sekar Kathiresan <skathiresan@vervetx.com>; Padma Malyala <pmalyala@vervetx.com>; Kallanthottathil Rajeev <krajeev@vervetx.com>; Andrew Bellinger <abellinger@vervetx.com>; sowmyai@bu.edu; Yuri Matsumoto <ymatsumoto@vervetx.com>
Subject: REPLY REQUESTED: Author change for GalNAc Manuscript NCOMMS-22-08508C
Importance: High

Dear Team:

Nature Communications requires all-author approval when the author list is changed. Dr. Yuri Matsumoto was mistakenly left off of the authors list and will thus be added as a correction. I will be collating these responses and providing them to *Nature Communications*.

Can you please each reply to this e-mail with the statement:

> **"As a coauthor of this manuscript, I agree with the addition of Dr. Yuri Matsumoto to the author list as outlined below."**

Updated byline is below.

GalNAc-Lipid nanoparticles enable non-LDLR dependent hepatic delivery of a CRISPR base editing therapy

Lisa N. Kasiewicz[1], Souvik Biswas[1], Aaron Beach[1], Huilan Ren[1], Chaitali Dutta[1], Anne Marie Mazzola[1], Ellen Rohde[1], Alexandra Chadwick[1], Christopher Cheng[1], Sara P. Garcia[1],
Sowmya Iyer[1], **Yuri Matsumoto[1]**, Amit V. Khera[1], Kiran Musunuru[2], Sekar Kathiresan[1], Padma Malyala[1], Kallanthottathil G. Rajeev[1], and Andrew M. Bellinger[1]*

Thank you very much for your assistance,
Lisa

Lisa Kasiewicz

From: Chaitali Dutta
Sent: Wednesday, March 1, 2023 4:29 PM
To: Lisa Kasiewicz
Subject: RE: REPLY REQUESTED: Author change for GalNAc Manuscript NCOMMS-22-08508C

As a coauthor of this manuscript, I agree with the addition of Dr. Yuri Matsumoto to the author list as outlined below.

GalNAc-Lipid nanoparticles enable non-LDLR dependent hepatic delivery of a CRISPR base editing therapy

Regards,
Chaitali

From: Lisa Kasiewicz <lkasiewicz@vervetx.com>
Sent: Wednesday, March 1, 2023 3:09 PM
To: Souvik Biswas <sbiswas@vervetx.com>; Aaron Beach <abeach@vervetx.com>; Huilan Ren <hren@vervetx.com>; Chaitali Dutta <cdutta@vervetx.com>; Anne Marie Mazzola <ammazzola@vervetx.com>; Ellen Rohde <erohde@vervetx.com>; Alexandra Chadwick <achadwick@vervetx.com>; Christopher Cheng <ccheng@vervetx.com>; Sara Garcia <sgarcia@vervetx.com>; Amit Khera <akhera@vervetx.com>; Kiran Musunuru <kmusunuru@vervetx.com>; Sekar Kathiresan <skathiresan@vervetx.com>; Padma Malyala <pmalyala@vervetx.com>; Kallanthottathil Rajeev <krajeev@vervetx.com>; Andrew Bellinger <abellinger@vervetx.com>; sowmyai@bu.edu; Yuri Matsumoto <ymatsumoto@vervetx.com>
Subject: REPLY REQUESTED: Author change for GalNAc Manuscript NCOMMS-22-08508C
Importance: High

Dear Team:

Nature Communications requires all-author approval when the author list is changed. Dr. Yuri Matsumoto was mistakenly left off of the authors list and will thus be added as a correction. I will be collating these responses and providing them to *Nature Communications*.

Can you please each reply to this e-mail with the statement:

> **"As a coauthor of this manuscript, I agree with the addition of Dr. Yuri Matsumoto to the author list as outlined below."**

Updated byline is below.

GalNAc-Lipid nanoparticles enable non-LDLR dependent hepatic delivery of a CRISPR base editing therapy

Lisa N. Kasiewicz[1], Souvik Biswas[1], Aaron Beach[1], Huilan Ren[1], Chaitali Dutta[1], Anne Marie Mazzola[1], Ellen Rohde[1], Alexandra Chadwick[1], Christopher Cheng[1], Sara P. Garcia[1], Sowmya Iyer[1], **Yuri Matsumoto**[1], Amit V. Khera[1], Kiran Musunuru[2], Sekar Kathiresan[1], Padma Malyala[1], Kallanthottathil G. Rajeev[1], and Andrew M. Bellinger[1]*

Thank you very much for your assistance,
Lisa

Lisa Kasiewicz

From: Anne Marie Mazzola
Sent: Wednesday, March 1, 2023 3:10 PM
To: Lisa Kasiewicz
Subject: RE: REPLY REQUESTED: Author change for GalNAc Manuscript NCOMMS-22-08508C

As a coauthor of this manuscript, I agree with the addition of Dr. Yuri Matsumoto to the author list as outlined below.

GalNAc-Lipid nanoparticles enable non-LDLR dependent hepatic delivery of a CRISPR base editing therapy

Lisa N. Kasiewicz[1], Souvik Biswas[1], Aaron Beach[1], Huilan Ren[1], Chaitali Dutta[1], Anne Marie Mazzola[1], Ellen Rohde[1], Alexandra Chadwick[1], Christopher Cheng[1], Sara P. Garcia[1],
Sowmya Iyer[1], **Yuri Matsumoto**[1], Amit V. Khera[1], Kiran Musunuru[2], Sekar Kathiresan[1], Padma Malyala[1], Kallanthottathil G. Rajeev[1], and Andrew M. Bellinger[1*]

From: Lisa Kasiewicz <lkasiewicz@vervetx.com>
Sent: Wednesday, March 1, 2023 3:09 PM
To: Souvik Biswas <sbiswas@vervetx.com>; Aaron Beach <abeach@vervetx.com>; Huilan Ren <hren@vervetx.com>; Chaitali Dutta <cdutta@vervetx.com>; Anne Marie Mazzola <ammazzola@vervetx.com>; Ellen Rohde <erohde@vervetx.com>; Alexandra Chadwick <achadwick@vervetx.com>; Christopher Cheng <ccheng@vervetx.com>; Sara Garcia <sgarcia@vervetx.com>; Amit Khera <akhera@vervetx.com>; Kiran Musunuru <kmusunuru@vervetx.com>; Sekar Kathiresan <skathiresan@vervetx.com>; Padma Malyala <pmalyala@vervetx.com>; Kallanthottathil Rajeev <krajeev@vervetx.com>; Andrew Bellinger <abellinger@vervetx.com>; sowmyai@bu.edu; Yuri Matsumoto <ymatsumoto@vervetx.com>
Subject: REPLY REQUESTED: Author change for GalNAc Manuscript NCOMMS-22-08508C
Importance: High

Dear Team:

Nature Communications requires all-author approval when the author list is changed. Dr. Yuri Matsumoto was mistakenly left off of the authors list and will thus be added as a correction. I will be collating these responses and providing them to *Nature Communications*.

Can you please each reply to this e-mail with the statement:

> **"As a coauthor of this manuscript, I agree with the addition of Dr. Yuri Matsumoto to the author list as outlined below."**

Updated byline is below.

GalNAc-Lipid nanoparticles enable non-LDLR dependent hepatic delivery of a CRISPR base editing therapy

Lisa N. Kasiewicz[1], Souvik Biswas[1], Aaron Beach[1], Huilan Ren[1], Chaitali Dutta[1], Anne Marie Mazzola[1], Ellen Rohde[1], Alexandra Chadwick[1], Christopher Cheng[1], Sara P. Garcia[1],
Sowmya Iyer[1], **Yuri Matsumoto**[1], Amit V. Khera[1], Kiran Musunuru[2], Sekar Kathiresan[1], Padma Malyala[1], Kallanthottathil G. Rajeev[1], and Andrew M. Bellinger[1*]

Thank you very much for your assistance,
Lisa

Lisa Kasiewicz

From: Ellen Rohde
Sent: Wednesday, March 1, 2023 8:50 PM
To: Lisa Kasiewicz
Subject: RE: REPLY REQUESTED: Author change for GalNAc Manuscript NCOMMS-22-08508C

Hi Lisa,
As a coauthor of this manuscript, I agree with the addition of Dr. Yuri Matsumoto to the author list as outlined below.
Ellen Rohde

From: Lisa Kasiewicz <lkasiewicz@vervetx.com>
Sent: Wednesday, March 1, 2023 3:09 PM
To: Souvik Biswas <sbiswas@vervetx.com>; Aaron Beach <abeach@vervetx.com>; Huilan Ren <hren@vervetx.com>; Chaitali Dutta <cdutta@vervetx.com>; Anne Marie Mazzola <ammazzola@vervetx.com>; Ellen Rohde <erohde@vervetx.com>; Alexandra Chadwick <achadwick@vervetx.com>; Christopher Cheng <ccheng@vervetx.com>; Sara Garcia <sgarcia@vervetx.com>; Amit Khera <akhera@vervetx.com>; Kiran Musunuru <kmusunuru@vervetx.com>; Sekar Kathiresan <skathiresan@vervetx.com>; Padma Malyala <pmalyala@vervetx.com>; Kallanthottathil Rajeev <krajeev@vervetx.com>; Andrew Bellinger <abellinger@vervetx.com>; sowmyai@bu.edu; Yuri Matsumoto <ymatsumoto@vervetx.com>
Subject: REPLY REQUESTED: Author change for GalNAc Manuscript NCOMMS-22-08508C
Importance: High

Dear Team:

Nature Communications requires all-author approval when the author list is changed. Dr. Yuri Matsumoto was mistakenly left off of the authors list and will thus be added as a correction. I will be collating these responses and providing them to *Nature Communications*.

Can you please each reply to this e-mail with the statement:

> **"As a coauthor of this manuscript, I agree with the addition of Dr. Yuri Matsumoto to the author list as outlined below."**

Updated byline is below.

GalNAc-Lipid nanoparticles enable non-LDLR dependent hepatic delivery of a CRISPR base editing therapy

Lisa N. Kasiewicz[1], Souvik Biswas[1], Aaron Beach[1], Huilan Ren[1], Chaitali Dutta[1], Anne Marie Mazzola[1], Ellen Rohde[1], Alexandra Chadwick[1], Christopher Cheng[1], Sara P. Garcia[1],
Sowmya Iyer[1], **Yuri Matsumoto**[1], Amit V. Khera[1], Kiran Musunuru[2], Sekar Kathiresan[1], Padma Malyala[1], Kallanthottathil G. Rajeev[1], and Andrew M. Bellinger[1]*

Thank you very much for your assistance,
Lisa

Lisa Kasiewicz

From: Alexandra Chadwick
Sent: Wednesday, March 1, 2023 4:54 PM
To: Lisa Kasiewicz
Subject: Re: REPLY REQUESTED: Author change for GalNAc Manuscript NCOMMS-22-08508C

As a coauthor of this manuscript, I agree with the addition of Dr. Yuri Matsumoto to the author list as outlined below.

Alexandra Chadwick

From: Lisa Kasiewicz <lkasiewicz@vervetx.com>
Date: Wednesday, March 1, 2023 at 3:08 PM
To: Souvik Biswas <sbiswas@vervetx.com>, Aaron Beach <abeach@vervetx.com>, Huilan Ren <hren@vervetx.com>, Chaitali Dutta <cdutta@vervetx.com>, Anne Marie Mazzola <ammazzola@vervetx.com>, Ellen Rohde <erohde@vervetx.com>, Alexandra Chadwick <achadwick@vervetx.com>, Christopher Cheng <ccheng@vervetx.com>, Sara Garcia <sgarcia@vervetx.com>, Amit Khera <akhera@vervetx.com>, Kiran Musunuru <kmusunuru@vervetx.com>, Sekar Kathiresan <skathiresan@vervetx.com>, Padma Malyala <pmalyala@vervetx.com>, Kallanthottathil Rajeev <krajeev@vervetx.com>, Andrew Bellinger <abellinger@vervetx.com>, sowmyai@bu.edu <sowmyai@bu.edu>, Yuri Matsumoto <ymatsumoto@vervetx.com>
Subject: REPLY REQUESTED: Author change for GalNAc Manuscript NCOMMS-22-08508C

Dear Team:

Nature Communications requires all-author approval when the author list is changed. Dr. Yuri Matsumoto was mistakenly left off of the authors list and will thus be added as a correction. I will be collating these responses and providing them to *Nature Communications*.

Can you please each reply to this e-mail with the statement:

> **"As a coauthor of this manuscript, I agree with the addition of Dr. Yuri Matsumoto to the author list as outlined below."**

Updated byline is below.

GalNAc-Lipid nanoparticles enable non-LDLR dependent hepatic delivery of a CRISPR base editing therapy

Lisa N. Kasiewicz[1], Souvik Biswas[1], Aaron Beach[1], Huilan Ren[1], Chaitali Dutta[1], Anne Marie Mazzola[1], Ellen Rohde[1], Alexandra Chadwick[1], Christopher Cheng[1], Sara P. Garcia[1],
Sowmya Iyer[1], **Yuri Matsumoto**[1], Amit V. Khera[1], Kiran Musunuru[2], Sekar Kathiresan[1], Padma Malyala[1], Kallanthottathil G. Rajeev[1], and Andrew M. Bellinger[1*]

Thank you very much for your assistance,
Lisa

Lisa Kasiewicz

From: Christopher Cheng
Sent: Wednesday, March 1, 2023 7:15 PM
To: Andrew Bellinger; Lisa Kasiewicz; Souvik Biswas; Aaron Beach; Huilan Ren; Chaitali Dutta; Anne Marie Mazzola; Ellen Rohde; Alexandra Chadwick; Sara Garcia; Amit Khera; Kiran Musunuru; Sekar Kathiresan; Padma Malyala; Kallanthottathil Rajeev; sowmyai@bu.edu; Yuri Matsumoto
Subject: Re: REPLY REQUESTED: Author change for GalNAc Manuscript NCOMMS-22-08508C

As a coauthor of this manuscript, I agree with the addition of Dr. Yuri Matsumoto to the author list as outlined below.

Christopher Cheng

From: Andrew Bellinger <abellinger@vervetx.com>
Sent: Wednesday, March 1, 2023 6:59 PM
To: Lisa Kasiewicz <lkasiewicz@vervetx.com>; Souvik Biswas <sbiswas@vervetx.com>; Aaron Beach <abeach@vervetx.com>; Huilan Ren <hren@vervetx.com>; Chaitali Dutta <cdutta@vervetx.com>; Anne Marie Mazzola <ammazzola@vervetx.com>; Ellen Rohde <erohde@vervetx.com>; Alexandra Chadwick <achadwick@vervetx.com>; Christopher Cheng <ccheng@vervetx.com>; Sara Garcia <sgarcia@vervetx.com>; Amit Khera <akhera@vervetx.com>; Kiran Musunuru <kmusunuru@vervetx.com>; Sekar Kathiresan <skathiresan@vervetx.com>; Padma Malyala <pmalyala@vervetx.com>; Kallanthottathil Rajeev <krajeev@vervetx.com>; sowmyai@bu.edu <sowmyai@bu.edu>; Yuri Matsumoto <ymatsumoto@vervetx.com>
Subject: Re: REPLY REQUESTED: Author change for GalNAc Manuscript NCOMMS-22-08508C

As a coauthor of this manuscript, I agree with the addition of Dr. Yuri Matsumoto to the author list as outlined below.

Andrew Bellinger

From: Lisa Kasiewicz <lkasiewicz@vervetx.com>
Date: Wednesday, March 1, 2023 at 3:08 PM
To: Souvik Biswas <sbiswas@vervetx.com>, Aaron Beach <abeach@vervetx.com>, Huilan Ren <hren@vervetx.com>, Chaitali Dutta <cdutta@vervetx.com>, Anne Marie Mazzola <ammazzola@vervetx.com>, Ellen Rohde <erohde@vervetx.com>, Alexandra Chadwick <achadwick@vervetx.com>, Christopher Cheng <ccheng@vervetx.com>, Sara Garcia <sgarcia@vervetx.com>, Amit Khera <akhera@vervetx.com>, Kiran Musunuru <kmusunuru@vervetx.com>, Sekar Kathiresan <skathiresan@vervetx.com>, Padma Malyala <pmalyala@vervetx.com>, Kallanthottathil Rajeev <krajeev@vervetx.com>, Andrew Bellinger <abellinger@vervetx.com>, sowmyai@bu.edu <sowmyai@bu.edu>, Yuri Matsumoto <ymatsumoto@vervetx.com>
Subject: REPLY REQUESTED: Author change for GalNAc Manuscript NCOMMS-22-08508C

Dear Team:

Nature Communications requires all-author approval when the author list is changed. Dr. Yuri Matsumoto was mistakenly left off of the authors list and will thus be added as a correction. I will be collating these responses and providing them to *Nature Communications*.

Can you please each reply to this e-mail with the statement:

"As a coauthor of this manuscript, I agree with the addition of Dr. Yuri Matsumoto to the author list as outlined below."

Updated byline is below.

GalNAc-Lipid nanoparticles enable non-LDLR dependent hepatic delivery of a CRISPR base editing therapy

Lisa N. Kasiewicz[1], Souvik Biswas[1], Aaron Beach[1], Huilan Ren[1], Chaitali Dutta[1], Anne Marie Mazzola[1], Ellen Rohde[1], Alexandra Chadwick[1], Christopher Cheng[1], Sara P. Garcia[1],
Sowmya Iyer[1], **Yuri Matsumoto**[1], Amit V. Khera[1], Kiran Musunuru[2], Sekar Kathiresan[1], Padma Malyala[1], Kallanthottathil G. Rajeev[1], and Andrew M. Bellinger[1]*

Thank you very much for your assistance,
Lisa

Lisa Kasiewicz, PhD
Associate Principal Scientist, Formulation

verve THERAPEUTICS

O: (617) 603-0070 **M:** (203) 673-9730
E: lkasiewicz@vervetx.com
A: 201 Brookline Avenue, Suite 601| Boston, MA 02215
www.vervetx.com

CONFIDENTIALITY NOTICE: The information contained in this communication from the sender is confidential. It is intended solely for use by the recipient and others authorized to receive it. If you are not the recipient, you are hereby notified that any disclosure, copying, distribution or taking action in relation of the contents of this information is strictly prohibited and may be unlawful.

Lisa Kasiewicz

**From:**	Sara Garcia
**Sent:**	Wednesday, March 1, 2023 4:47 PM
**To:**	Aaron Beach; Lisa Kasiewicz; Souvik Biswas; Huilan Ren; Chaitali Dutta; Anne Marie Mazzola; Ellen Rohde; Alexandra Chadwick; Christopher Cheng; Amit Khera; Kiran Musunuru; Sekar Kathiresan; Padma Malyala; Kallanthottathil Rajeev; Andrew Bellinger; sowmyai@bu.edu; Yuri Matsumoto
**Subject:**	Re: REPLY REQUESTED: Author change for GalNAc Manuscript NCOMMS-22-08508C

As a coauthor of this manuscript, I agree with the addition of Dr. Yuri Matsumoto to the author list as outlined below.

Sara Garcia

From: Aaron Beach <abeach@vervetx.com>
Date: Wednesday, March 1, 2023 at 4:42 PM
To: Lisa Kasiewicz <lkasiewicz@vervetx.com>, Souvik Biswas <sbiswas@vervetx.com>, Huilan Ren <hren@vervetx.com>, Chaitali Dutta <cdutta@vervetx.com>, Anne Marie Mazzola <ammazzola@vervetx.com>, Ellen Rohde <erohde@vervetx.com>, Alexandra Chadwick <achadwick@vervetx.com>, Christopher Cheng <ccheng@vervetx.com>, Sara Garcia <sgarcia@vervetx.com>, Amit Khera <akhera@vervetx.com>, Kiran Musunuru <kmusunuru@vervetx.com>, Sekar Kathiresan <skathiresan@vervetx.com>, Padma Malyala <pmalyala@vervetx.com>, Kallanthottathil Rajeev <krajeev@vervetx.com>, Andrew Bellinger <abellinger@vervetx.com>, sowmyai@bu.edu <sowmyai@bu.edu>, Yuri Matsumoto <ymatsumoto@vervetx.com>
Subject: RE: REPLY REQUESTED: Author change for GalNAc Manuscript NCOMMS-22-08508C

As a coauthor of this manuscript, I agree with the addition of Dr. Yuri Matsumoto to the author list as outlined below.

GalNAc-Lipid nanoparticles enable non-LDLR dependent hepatic delivery of a CRISPR base editing therapy

Aaron Beach (he/his)
Senior Director – Internal Quality Control

O: (937) 287-6157 **M:** (937) 287-6157
E: abeach@vervetx.com
A: 201 Brookline Avenue, Suite 601, Boston, MA 02215
www.vervetx.com

CONFIDENTIALITY NOTICE: The information contained in this communication from the sender is confidential. It is intended solely for use by the recipient and others authorized to receive it. If you are not the recipient, you are hereby notified that any disclosure, copying, distribution or taking action in relation of the contents of this information is strictly prohibited and may be unlawful.

From: Lisa Kasiewicz <lkasiewicz@vervetx.com>
Sent: Wednesday, March 1, 2023 3:09 PM
To: Souvik Biswas <sbiswas@vervetx.com>; Aaron Beach <abeach@vervetx.com>; Huilan Ren <hren@vervetx.com>; Chaitali Dutta <cdutta@vervetx.com>; Anne Marie Mazzola <ammazzola@vervetx.com>; Ellen Rohde <erohde@vervetx.com>; Alexandra Chadwick <achadwick@vervetx.com>; Christopher Cheng <ccheng@vervetx.com>;

Sara Garcia <sgarcia@vervetx.com>; Amit Khera <akhera@vervetx.com>; Kiran Musunuru <kmusunuru@vervetx.com>; Sekar Kathiresan <skathiresan@vervetx.com>; Padma Malyala <pmalyala@vervetx.com>; Kallanthottathil Rajeev <krajeev@vervetx.com>; Andrew Bellinger <abellinger@vervetx.com>; sowmyai@bu.edu; Yuri Matsumoto <ymatsumoto@vervetx.com>
Subject: REPLY REQUESTED: Author change for GalNAc Manuscript NCOMMS-22-08508C
Importance: High

Dear Team:

Nature Communications requires all-author approval when the author list is changed. Dr. Yuri Matsumoto was mistakenly left off of the authors list and will thus be added as a correction. I will be collating these responses and providing them to *Nature Communications*.

Can you please each reply to this e-mail with the statement:

> **"As a coauthor of this manuscript, I agree with the addition of Dr. Yuri Matsumoto to the author list as outlined below."**

Updated byline is below.

GalNAc-Lipid nanoparticles enable non-LDLR dependent hepatic delivery of a CRISPR base editing therapy

Lisa N. Kasiewicz[1], Souvik Biswas[1], Aaron Beach[1], Huilan Ren[1], Chaitali Dutta[1], Anne Marie Mazzola[1], Ellen Rohde[1], Alexandra Chadwick[1], Christopher Cheng[1], Sara P. Garcia[1],
Sowmya Iyer[1], **Yuri Matsumoto**[1], Amit V. Khera[1], Kiran Musunuru[2], Sekar Kathiresan[1], Padma Malyala[1], Kallanthottathil G. Rajeev[1], and Andrew M. Bellinger[1*]

Thank you very much for your assistance,
Lisa

Lisa Kasiewicz, PhD
Associate Principal Scientist, Formulation

verve THERAPEUTICS

O: (617) 603-0070 **M:** (203) 673-9730
E: lkasiewicz@vervetx.com
A: 201 Brookline Avenue, Suite 601| Boston, MA 02215
www.vervetx.com

CONFIDENTIALITY NOTICE: The information contained in this communication from the sender is confidential. It is intended solely for use by the recipient and others authorized to receive it. If you are not the recipient, you are hereby notified that any disclosure, copying, distribution or taking action in relation of the contents of this information is strictly prohibited and may be unlawful.

Lisa Kasiewicz

**From:**	Sowmya Iyer <sowmyai@bu.edu>
**Sent:**	Wednesday, March 1, 2023 3:38 PM
**To:**	Lisa Kasiewicz
**Cc:**	Aaron Beach; Alexandra Chadwick; Amit Khera; Andrew Bellinger; Anne Marie Mazzola; Chaitali Dutta; Christopher Cheng; Ellen Rohde; Huilan Ren; Kallanthottathil Rajeev; Kiran Musunuru; Padma Malyala; Sara Garcia; Sekar Kathiresan; Souvik Biswas; Yuri Matsumoto
**Subject:**	Re: REPLY REQUESTED: Author change for GalNAc Manuscript NCOMMS-22-08508C

As a coauthor of this manuscript, I agree with the addition of Dr. Yuri Matsumoto to the author list as outlined below.

Thanks,
Sowmya Iyer

On Wed, Mar 1, 2023 at 3:09 PM Lisa Kasiewicz <lkasiewicz@vervetx.com> wrote:

Dear Team:

Nature Communications requires all-author approval when the author list is changed. Dr. Yuri Matsumoto was mistakenly left off of the authors list and will thus be added as a correction. I will be collating these responses and providing them to *Nature Communications*.

Can you please each reply to this e-mail with the statement:

> **"As a coauthor of this manuscript, I agree with the addition of Dr. Yuri Matsumoto to the author list as outlined below."**

Updated byline is below.

GalNAc-Lipid nanoparticles enable non-LDLR dependent hepatic delivery of a CRISPR base editing therapy

Lisa N. Kasiewicz[1], Souvik Biswas[1], Aaron Beach[1], Huilan Ren[1], Chaitali Dutta[1], Anne Marie Mazzola[1], Ellen Rohde[1], Alexandra Chadwick[1], Christopher Cheng[1], Sara P. Garcia[1],

Sowmya Iyer[1], **Yuri Matsumoto**[1], Amit V. Khera[1], Kiran Musunuru[2], Sekar Kathiresan[1], Padma Malyala[1], Kallanthottathil G. Rajeev[1], and Andrew M. Bellinger[1]*

Thank you very much for your assistance,

Lisa Kasiewicz

**From:**	Amit Khera
**Sent:**	Wednesday, March 1, 2023 3:09 PM
**To:**	Lisa Kasiewicz
**Subject:**	Re: REPLY REQUESTED: Author change for GalNAc Manuscript NCOMMS-22-08508C

"As a coauthor of this manuscript, I agree with the addition of Dr. Yuri Matsumoto to the author list as outlined below."

From: Lisa Kasiewicz <lkasiewicz@vervetx.com>
Date: Wednesday, March 1, 2023 at 3:08 PM
To: Souvik Biswas <sbiswas@vervetx.com>, Aaron Beach <abeach@vervetx.com>, Huilan Ren <hren@vervetx.com>, Chaitali Dutta <cdutta@vervetx.com>, Anne Marie Mazzola <ammazzola@vervetx.com>, Ellen Rohde <erohde@vervetx.com>, Alexandra Chadwick <achadwick@vervetx.com>, Christopher Cheng <ccheng@vervetx.com>, Sara Garcia <sgarcia@vervetx.com>, Amit Khera <akhera@vervetx.com>, Kiran Musunuru <kmusunuru@vervetx.com>, Sekar Kathiresan <skathiresan@vervetx.com>, Padma Malyala <pmalyala@vervetx.com>, Kallanthottathil Rajeev <krajeev@vervetx.com>, Andrew Bellinger <abellinger@vervetx.com>, sowmyai@bu.edu <sowmyai@bu.edu>, Yuri Matsumoto <ymatsumoto@vervetx.com>
Subject: REPLY REQUESTED: Author change for GalNAc Manuscript NCOMMS-22-08508C

Dear Team:

Nature Communications requires all-author approval when the author list is changed. Dr. Yuri Matsumoto was mistakenly left off of the authors list and will thus be added as a correction. I will be collating these responses and providing them to *Nature Communications*.

Can you please each reply to this e-mail with the statement:

> **"As a coauthor of this manuscript, I agree with the addition of Dr. Yuri Matsumoto to the author list as outlined below."**

Updated byline is below.

GalNAc-Lipid nanoparticles enable non-LDLR dependent hepatic delivery of a CRISPR base editing therapy

Lisa N. Kasiewicz[1], Souvik Biswas[1], Aaron Beach[1], Huilan Ren[1], Chaitali Dutta[1], Anne Marie Mazzola[1], Ellen Rohde[1], Alexandra Chadwick[1], Christopher Cheng[1], Sara P. Garcia[1],
Sowmya Iyer[1], **Yuri Matsumoto**[1], Amit V. Khera[1], Kiran Musunuru[2], Sekar Kathiresan[1], Padma Malyala[1], Kallanthottathil G. Rajeev[1], and Andrew M. Bellinger[1]*

Thank you very much for your assistance,
Lisa

Lisa Kasiewicz

From: Kiran Musunuru
Sent: Wednesday, March 1, 2023 7:59 PM
To: Lisa Kasiewicz
Subject: Re: REPLY REQUESTED: Author change for GalNAc Manuscript NCOMMS-22-08508C

As a coauthor of this manuscript, I agree with the addition of Dr. Yuri Matsumoto to the author list as outlined below.

Kiran Musunuru

CONFIDENTIALITY NOTICE: The information contained in this communication from the sender is confidential. It is intended solely for use by the recipient and others authorized to receive it. If you are not the recipient, you are hereby notified that any disclosure, copying, distribution or taking action in relation of the contents of this information is strictly prohibited and may be unlawful.

From: Lisa Kasiewicz <lkasiewicz@vervetx.com>
Sent: Wednesday, March 1, 2023 3:08 PM
To: Souvik Biswas <sbiswas@vervetx.com>; Aaron Beach <abeach@vervetx.com>; Huilan Ren <hren@vervetx.com>; Chaitali Dutta <cdutta@vervetx.com>; Anne Marie Mazzola <ammazzola@vervetx.com>; Ellen Rohde <erohde@vervetx.com>; Alexandra Chadwick <achadwick@vervetx.com>; Christopher Cheng <ccheng@vervetx.com>; Sara Garcia <sgarcia@vervetx.com>; Amit Khera <akhera@vervetx.com>; Kiran Musunuru <kmusunuru@vervetx.com>; Sekar Kathiresan <skathiresan@vervetx.com>; Padma Malyala <pmalyala@vervetx.com>; Kallanthottathil Rajeev <krajeev@vervetx.com>; Andrew Bellinger <abellinger@vervetx.com>; sowmyai@bu.edu <sowmyai@bu.edu>; Yuri Matsumoto <ymatsumoto@vervetx.com>
Subject: REPLY REQUESTED: Author change for GalNAc Manuscript NCOMMS-22-08508C

Dear Team:

Nature Communications requires all-author approval when the author list is changed. Dr. Yuri Matsumoto was mistakenly left off of the authors list and will thus be added as a correction. I will be collating these responses and providing them to *Nature Communications*.

Can you please each reply to this e-mail with the statement:

> **"As a coauthor of this manuscript, I agree with the addition of Dr. Yuri Matsumoto to the author list as outlined below."**

Updated byline is below.

GalNAc-Lipid nanoparticles enable non-LDLR dependent hepatic delivery of a CRISPR base editing therapy

Lisa N. Kasiewicz[1], Souvik Biswas[1], Aaron Beach[1], Huilan Ren[1], Chaitali Dutta[1], Anne Marie Mazzola[1], Ellen Rohde[1], Alexandra Chadwick[1], Christopher Cheng[1], Sara P. Garcia[1],
Sowmya Iyer[1], **Yuri Matsumoto**[1], Amit V. Khera[1], Kiran Musunuru[2], Sekar Kathiresan[1], Padma Malyala[1], Kallanthottathil G. Rajeev[1], and Andrew M. Bellinger[1]*

Thank you very much for your assistance,
Lisa

Lisa Kasiewicz

From: Sekar Kathiresan
Sent: Thursday, March 2, 2023 6:58 AM
To: Lisa Kasiewicz
Subject: Re: REPLY REQUESTED: Author change for GalNAc Manuscript NCOMMS-22-08508C

Dear Lisa,

"As a coauthor of this manuscript, I agree with the addition of Dr. Yuri Matsumoto to the author list as outlined below."

Best,
Sek

Sekar Kathiresan, MD
Chief Executive Officer

M: (617) 584-6343
E: skathiresan@vervetx.com
A: 201 Brookline Avenue, 6th Floor | Boston, MA 02215
www.vervetx.com

Learn more @nytimes here: A 'Cure for Heart Disease'? A Single Shot Succeeds in Monkeys

CONFIDENTIALITY NOTICE: The information contained in this communication from the sender is confidential. It is intended solely for use by the recipient and others authorized to receive it. If you are not the recipient, you are hereby notified that any disclosure, copying, distribution or taking action in relation of the contents of this information is strictly prohibited and may be unlawful.

From: Lisa Kasiewicz <lkasiewicz@vervetx.com>
Date: Wednesday, March 1, 2023 at 3:08 PM
To: Souvik Biswas <sbiswas@vervetx.com>, Aaron Beach <abeach@vervetx.com>, Huilan Ren <hren@vervetx.com>, Chaitali Dutta <cdutta@vervetx.com>, Anne Marie Mazzola <ammazzola@vervetx.com>, Ellen Rohde <erohde@vervetx.com>, Alexandra Chadwick <achadwick@vervetx.com>, Christopher Cheng <ccheng@vervetx.com>, Sara Garcia <sgarcia@vervetx.com>, Amit Khera <akhera@vervetx.com>, Kiran Musunuru <kmusunuru@vervetx.com>, Sekar Kathiresan <skathiresan@vervetx.com>, Padma Malyala <pmalyala@vervetx.com>, Kallanthottathill Rajeev <krajeev@vervetx.com>, Andrew Bellinger <abellinger@vervetx.com>, "sowmyai@bu.edu" <sowmyai@bu.edu>, Yuri Matsumoto <ymatsumoto@vervetx.com>
Subject: REPLY REQUESTED: Author change for GalNAc Manuscript NCOMMS-22-08508C

Dear Team:

Nature Communications requires all-author approval when the author list is changed. Dr. Yuri Matsumoto was mistakenly left off of the authors list and will thus be added as a correction. I will be collating these responses and providing them to *Nature Communications*.

Can you please each reply to this e-mail with the statement:

"As a coauthor of this manuscript, I agree with the addition of Dr. Yuri Matsumoto to the author list as outlined below."

Updated byline is below.

GalNAc-Lipid nanoparticles enable non-LDLR dependent hepatic delivery of a CRISPR base editing therapy

Lisa N. Kasiewicz[1], Souvik Biswas[1], Aaron Beach[1], Huilan Ren[1], Chaitali Dutta[1], Anne Marie Mazzola[1], Ellen Rohde[1], Alexandra Chadwick[1], Christopher Cheng[1], Sara P. Garcia[1],
Sowmya Iyer[1], **Yuri Matsumoto**[1], Amit V. Khera[1], Kiran Musunuru[2], Sekar Kathiresan[1], Padma Malyala[1], Kallanthottathil G. Rajeev[1], and Andrew M. Bellinger[1]*

Thank you very much for your assistance,
Lisa

Lisa Kasiewicz, PhD
Associate Principal Scientist, Formulation

verve THERAPEUTICS

O: (617) 603-0070 **M:** (203) 673-9730
E: lkasiewicz@vervetx.com
A: 201 Brookline Avenue, Suite 601| Boston, MA 02215
www.vervetx.com

CONFIDENTIALITY NOTICE: The information contained in this communication from the sender is confidential. It is intended solely for use by the recipient and others authorized to receive it. If you are not the recipient, you are hereby notified that any disclosure, copying, distribution or taking action in relation of the contents of this information is strictly prohibited and may be unlawful.

Lisa Kasiewicz

From: Padma Malyala
Sent: Thursday, March 2, 2023 10:18 AM
To: Lisa Kasiewicz; Souvik Biswas; Aaron Beach; Huilan Ren; Chaitali Dutta; Anne Marie Mazzola; Ellen Rohde; Alexandra Chadwick; Christopher Cheng; Sara Garcia; Amit Khera; Kiran Musunuru; Sekar Kathiresan; Kallanthottathil Rajeev; Andrew Bellinger; sowmyai@bu.edu; Yuri Matsumoto
Subject: RE: REPLY REQUESTED: Author change for GalNAc Manuscript NCOMMS-22-08508C

Hi,

As a coauthor of this manuscript, I agree with the addition of Dr. Yuri Matsumoto to the author list as outlined below."

Updated byline is below.

GalNAc-Lipid nanoparticles enable non-LDLR dependent hepatic delivery of a CRISPR base editing therapy

Lisa N. Kasiewicz[1], Souvik Biswas[1], Aaron Beach[1], Huilan Ren[1], Chaitali Dutta[1], Anne Marie Mazzola[1], Ellen Rohde[1], Alexandra Chadwick[1], Christopher Cheng[1], Sara P. Garcia[1],
Sowmya Iyer[1], **Yuri Matsumoto[1]**, Amit V. Khera[1], Kiran Musunuru[2], Sekar Kathiresan[1], Padma Malyala[1], Kallanthottathil G. Rajeev[1], and Andrew M. Bellinger[1]*

Warm Regards,
Padma

Padma Malyala
Vice President, Formulation & Analytical Development

Verve Therapeutics
O: (617) 603-0070 **M:** (857) 208-3848
E: pmalyala@vervetx.com
A: 201 BrooklineAvenue, 6th Floor| Boston, MA 02215
www.vervetx.com

CONFIDENTIALITY NOTICE: The information contained in this communication from the sender is confidential. It is intended solely for use by the recipient and others authorized to receive it. If you are not the recipient, you are hereby notified that any disclosure, copying, distribution or taking action in relation of the contents of this information is strictly prohibited and may be unlawful.

From: Lisa Kasiewicz <lkasiewicz@vervetx.com>
Sent: Wednesday, March 1, 2023 3:09 PM
To: Souvik Biswas <sbiswas@vervetx.com>; Aaron Beach <abeach@vervetx.com>; Huilan Ren <hren@vervetx.com>; Chaitali Dutta <cdutta@vervetx.com>; Anne Marie Mazzola <ammazzola@vervetx.com>; Ellen Rohde <erohde@vervetx.com>; Alexandra Chadwick <achadwick@vervetx.com>; Christopher Cheng <ccheng@vervetx.com>; Sara Garcia <sgarcia@vervetx.com>; Amit Khera <akhera@vervetx.com>; Kiran Musunuru <kmusunuru@vervetx.com>; Sekar Kathiresan <skathiresan@vervetx.com>; Padma Malyala <pmalyala@vervetx.com>; Kallanthottathil Rajeev <krajeev@vervetx.com>; Andrew Bellinger <abellinger@vervetx.com>; sowmyai@bu.edu; Yuri Matsumoto

Lisa Kasiewicz

From: Kallanthottathil Rajeev
Sent: Wednesday, March 1, 2023 4:37 PM
To: Lisa Kasiewicz; Souvik Biswas; Aaron Beach; Huilan Ren; Chaitali Dutta; Anne Marie Mazzola; Ellen Rohde; Alexandra Chadwick; Christopher Cheng; Sara Garcia; Amit Khera; Kiran Musunuru; Sekar Kathiresan; Padma Malyala; Andrew Bellinger; sowmyai@bu.edu; Yuri Matsumoto
Subject: Re: REPLY REQUESTED: Author change for GalNAc Manuscript NCOMMS-22-08508C

As a coauthor of this manuscript, I agree with the addition of Dr. Yuri Matsumoto to the author list as outlined below.
Rajeev

Kallanthottathil Rajeev (Rajeev)
Vice President, CMC
O: (617) 603-0070 **M:** (617) 331-7618
E: krajeev@vervetx.com
A: 201 Brookline Avenue, Suite 601, Boston, MA 02215
www.vervetx.com

The information contained in this communication from the sender is confidential. It is intended solely for use by the recipient and others authorized to receive it. If you are not the recipient, you are hereby notified that any disclosure, copying, distribution or taking action in relation of the contents of this information is strictly prohibited and may be unlawful.

From: Lisa Kasiewicz <lkasiewicz@vervetx.com>
Date: Wednesday, March 1, 2023 at 3:08 PM
To: Souvik Biswas <sbiswas@vervetx.com>, Aaron Beach <abeach@vervetx.com>, Huilan Ren <hren@vervetx.com>, Chaitali Dutta <cdutta@vervetx.com>, Anne Marie Mazzola <ammazzola@vervetx.com>, Ellen Rohde <erohde@vervetx.com>, Alexandra Chadwick <achadwick@vervetx.com>, Christopher Cheng <ccheng@vervetx.com>, Sara Garcia <sgarcia@vervetx.com>, Amit Khera <akhera@vervetx.com>, Kiran Musunuru <kmusunuru@vervetx.com>, Sekar Kathiresan <skathiresan@vervetx.com>, Padma Malyala <pmalyala@vervetx.com>, Kallanthottathil Rajeev <krajeev@vervetx.com>, Andrew Bellinger <abellinger@vervetx.com>, sowmyai@bu.edu <sowmyai@bu.edu>, Yuri Matsumoto <ymatsumoto@vervetx.com>
Subject: REPLY REQUESTED: Author change for GalNAc Manuscript NCOMMS-22-08508C

Dear Team:

Nature Communications requires all-author approval when the author list is changed. Dr. Yuri Matsumoto was mistakenly left off of the authors list and will thus be added as a correction. I will be collating these responses and providing them to *Nature Communications*.

Can you please each reply to this e-mail with the statement:

> **"As a coauthor of this manuscript, I agree with the addition of Dr. Yuri Matsumoto to the author list as outlined below."**

Updated byline is below.

GalNAc-Lipid nanoparticles enable non-LDLR dependent hepatic delivery of a CRISPR base editing therapy

Lisa N. Kasiewicz[1], Souvik Biswas[1], Aaron Beach[1], Huilan Ren[1], Chaitali Dutta[1], Anne Marie Mazzola[1], Ellen Rohde[1], Alexandra Chadwick[1], Christopher Cheng[1], Sara P. Garcia[1],
Sowmya Iyer[1], **Yuri Matsumoto**[1], Amit V. Khera[1], Kiran Musunuru[2], Sekar Kathiresan[1], Padma Malyala[1], Kallanthottathil G. Rajeev[1], and Andrew M. Bellinger[1*]

Thank you very much for your assistance,
Lisa

Lisa Kasiewicz, PhD
Associate Principal Scientist, Formulation

O: (617) 603-0070 **M:** (203) 673-9730
E: lkasiewicz@vervetx.com
A: 201 Brookline Avenue, Suite 601| Boston, MA 02215
www.vervetx.com

CONFIDENTIALITY NOTICE: The information contained in this communication from the sender is confidential. It is intended solely for use by the recipient and others authorized to receive it. If you are not the recipient, you are hereby notified that any disclosure, copying, distribution or taking action in relation of the contents of this information is strictly prohibited and may be unlawful.

Lisa Kasiewicz

From: Andrew Bellinger
Sent: Wednesday, March 1, 2023 6:59 PM
To: Lisa Kasiewicz; Souvik Biswas; Aaron Beach; Huilan Ren; Chaitali Dutta; Anne Marie Mazzola; Ellen Rohde; Alexandra Chadwick; Christopher Cheng; Sara Garcia; Amit Khera; Kiran Musunuru; Sekar Kathiresan; Padma Malyala; Kallanthottathil Rajeev; sowmyai@bu.edu; Yuri Matsumoto
Subject: Re: REPLY REQUESTED: Author change for GalNAc Manuscript NCOMMS-22-08508C

As a coauthor of this manuscript, I agree with the addition of Dr. Yuri Matsumoto to the author list as outlined below.

Andrew Bellinger

From: Lisa Kasiewicz <lkasiewicz@vervetx.com>
Date: Wednesday, March 1, 2023 at 3:08 PM
To: Souvik Biswas <sbiswas@vervetx.com>, Aaron Beach <abeach@vervetx.com>, Huilan Ren <hren@vervetx.com>, Chaitali Dutta <cdutta@vervetx.com>, Anne Marie Mazzola <ammazzola@vervetx.com>, Ellen Rohde <erohde@vervetx.com>, Alexandra Chadwick <achadwick@vervetx.com>, Christopher Cheng <ccheng@vervetx.com>, Sara Garcia <sgarcia@vervetx.com>, Amit Khera <akhera@vervetx.com>, Kiran Musunuru <kmusunuru@vervetx.com>, Sekar Kathiresan <skathiresan@vervetx.com>, Padma Malyala <pmalyala@vervetx.com>, Kallanthottathil Rajeev <krajeev@vervetx.com>, Andrew Bellinger <abellinger@vervetx.com>, sowmyai@bu.edu <sowmyai@bu.edu>, Yuri Matsumoto <ymatsumoto@vervetx.com>
Subject: REPLY REQUESTED: Author change for GalNAc Manuscript NCOMMS-22-08508C

Dear Team:

Nature Communications requires all-author approval when the author list is changed. Dr. Yuri Matsumoto was mistakenly left off of the authors list and will thus be added as a correction. I will be collating these responses and providing them to *Nature Communications*.

Can you please each reply to this e-mail with the statement:

> **"As a coauthor of this manuscript, I agree with the addition of Dr. Yuri Matsumoto to the author list as outlined below."**

Updated byline is below.

GalNAc-Lipid nanoparticles enable non-LDLR dependent hepatic delivery of a CRISPR base editing therapy

Lisa N. Kasiewicz[1], Souvik Biswas[1], Aaron Beach[1], Huilan Ren[1], Chaitali Dutta[1], Anne Marie Mazzola[1], Ellen Rohde[1], Alexandra Chadwick[1], Christopher Cheng[1], Sara P. Garcia[1],
Sowmya Iyer[1], **Yuri Matsumoto**[1], Amit V. Khera[1], Kiran Musunuru[2], Sekar Kathiresan[1], Padma Malyala[1], Kallanthottathil G. Rajeev[1], and Andrew M. Bellinger[1*]

Thank you very much for your assistance,
Lisa
